# Novel Gold-Functionalization of Liposomes and Its Impact on Cellular Uptake and Trafficking

**DOI:** 10.1021/acs.molpharmaceut.5c01722

**Published:** 2026-04-10

**Authors:** Agata Margielewska, Klaudia Łuków, Abdelatif Laroui, Monika Marcinkowska, Sylwia Michlewska, Michał Gorzkiewicz, Łukasz Półtorak, Barbara Klajnert-Maculewicz

**Affiliations:** † 49602 University of Lodz, Faculty of Biology and Environmental Protection, Department of General Biophysics, 141/143 Pomorska St., Lodz 90-236, Poland; ‡ Bio-Med-Chem Doctoral School of the University of Lodz and Lodz Institutes of the Polish Academy of Sciences, University of Lodz, 12/16 Banacha St., Lodz 90-237, Poland; § University of Lodz Electrochemistry@Soft Interfaces (E@SI) Team, Department of Inorganic and Analytical Chemistry, Faculty of Chemistry, 12 Tamka St., Lodz 91-403, Poland; ∥ Doctoral School of Exact and Natural Sciences, University of Lodz, 12/16 Banacha St. Lodz 90-237, Poland; ⊥ 196812 University of Lodz, Faculty of Biology and Environmental Protection, Laboratory of Microscopic Imaging and Specialized Biological Techniques, 12/16 Banacha St. Lodz 90-237, Poland; # Department of Molecular Medicine II, Medical Faculty and University Hospital, Heinrich Heine University Düsseldorf, Universitätsstr. 1, Düsseldorf 40225, Germany

**Keywords:** phospholipids, AuNPs, cellular internalization, cytotoxicity, voltammetry, nanocarrier

## Abstract

Liposomes decorated with gold nanoparticles (AuNPs) represent multifunctional nanocarriers that combine high drug-loading capacity with the unique physicochemical properties of metallic nanoparticles. Here, we report a simple one-pot strategy for the controlled surface attachment of citrate-stabilized AuNPs to liposomes, based on the synergistic action of electrostatic and covalent interactions. This was achieved by incorporating two functional lipids: the cationic lipid DOTAP, providing a permanent positive surface charge that promotes electrostatic attraction toward negatively charged AuNPs, and the thiol-containing lipid DPSH, possessing a functional group known for stable Au–S bond formation. Simple mixing of AuNPs with DOTAP&DPSH liposomes resulted in a hydrodynamic diameter increase corresponding approximately to twice the AuNP diameter, a reduction in ζ-potential, and a red shift of the AuNP plasmon absorption maximum, confirming nanoparticle attachment. Adsorption of AuNPs onto lipid monolayers was further verified electrochemically at the electrified liquid–liquid interface. Biological studies demonstrated that AuNP decoration significantly enhanced cellular uptake. The effect was most pronounced after 24 h of incubation at a lipid concentration of 40 μg·mL^–1^ in HeLa cells, where fluorescence intensity increased by approximately 60% compared to nondecorated liposomes. Confocal colocalization analysis indicated a reduced level of trafficking of LipoAuNPs to LysoTracker-positive compartments, suggesting altered intracellular processing. All formulations showed negligible cytotoxicity within the tested concentration range. This study provides mechanistic insight into AuNP–liposome conjugation and demonstrates that dual electrostatic–covalent anchoring improves cellular internalization while maintaining biocompatibility.

## Graphical abstract

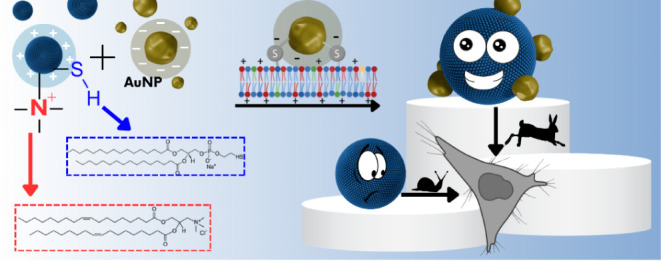

## 1 Introduction

Nanomedicine focuses on the development of advanced drug delivery systems that can effectively transport active substances of interest to the target site of action and then across the cell membrane. An ideal carrier should be characterized by (i) nontoxicity, (ii) biodegradability, (iii) targeting specificity, (iv) ease of preparation, (v) efficient encapsulation of active substances, (vi) controlled release, and (vii) scalability in manufacturing for clinical translation. Simultaneously, the carrier must traverse multiple biological barriers on its way to the site of action. The limited number of nanosystems that have been translated into clinical practice highlights the need for further development, correlated with a solid fundamental understanding of their properties.^1,2^ Undoubtedly, liposomes stand out as one of the few nanosystems that have successfully transitioned from the laboratory bench to medical applications. The enormous success of liposomes is due, among other factors, to their nontoxicity, biocompatibility, and high capacity to encapsulate both hydrophilic and hydrophobic compounds.^3,4^ Currently, diverse liposomal surface modification strategies are being investigated to tailor liposome properties, including active targeting and controlled drug release.

A very interesting and promising example of second-generation liposomes features surface modification with gold nanoparticles (AuNPs). These hybrid systems (LipoAuNPs) combine the advantageous features of both components: the encapsulation capacity of liposomes and the unique properties of AuNPs. Specifically, AuNPs possess unique optoelectrical properties due to their localized, particle-size-dependent plasmon resonance. Furthermore, they are characterized by strong absorption of electromagnetic waves and the ability to convert photon energy into thermal energy.^5–7^ As a result, they show tremendous potential for use in photothermal therapy (PTT) and thermal imaging.^5^ In PTT, AuNPs absorb electromagnetic radiation and increase their temperature faster than the surroundings. This allows for the rapid generation of local hyperthermia in the area of interest. In the field of imaging, the optoelectronic properties of AuNPs are tested as contrast agents in various techniques such as X-ray, computed tomography, multimodal imaging and photoacoustic imaging.^8,9^ The second crucial advantage of attaching AuNPs to the liposomal surface relates to their straightforward surface functionalization, which requires little to no energetic input.^10,11^ AuNPs are commonly surface-modified to enhance their colloidal stability, prevent aggregation, and restrict protein corona formation in biological media.^12–14^ Additionally, fine-tuning of AuNPs fabrication protocols allows precise control of their morphology, size, and shape.^15–17^ The most common shapes of AuNPs include nanospheres, nanorods, nanostars, nanocubes, and nanotriangles.^5,18^ Moreover, AuNPs exhibit minimal toxicity and immunogenicity and possess a proven ability to efficiently enter cells via endocytosis, which expands their biomedical potential.^19^ ^,^

Despite the many advantages and unique features of AuNPs, phase II or more advanced clinical trials of free AuNPs are rather limited. According to Badir et al.^20^ this is mainly due to the still not yet well-understood correlation between AuNP size, their clearance from the body, and their long-term safety: larger particles or those with nonbiodegradable surface coatings may accumulate in tissues and pose chronic health risks.^20^ Liposomes provide a biodegradable cargo for AuNPs, allowing for increased nanosystem size (advantageous for the enhanced permeability and retention effect). Additionally, since AuNPs on the liposome surface exhibit a different maximum of absorption compared to free AuNPs, simple adjustment of the formulation protocol can be used to control their spectroscopic properties.^21,22^ However, the combination of AuNPs and liposomes merges their characteristics while significantly reducing the disadvantages and limitations of both nanosystems. This feature is especially desired for biomedical applications. In the present study, we propose and biologically evaluate a rational strategy for attaching AuNPs to the surface of liposomes, representing a structurally and mechanistically distinct approach compared with previously reported liposome–AuNP systems.

Other liposome surface decoration protocols with AuNPs by *ex situ* methods (i.e., AuNPs are first synthesized and then attached to the liposomal surface) are described in the scientific literature, yet still await straightforward and comprehensively understood, reproducible attachment protocols. Our primary innovation lies in integrating two leading strategies for AuNP–liposome conjugation: covalent bonding between AuNPs and liposomes^23,24^ and electrostatic attraction of AuNPs and liposomes.^22,25–30^ While approaches relying solely on electrostatic attraction have been relatively widely investigated for biomedical applications, combining this strategy with covalent bonding brings many advantages:

**• Covalent bonding** provides stability and resistance to pH changes, reducing the risk of AuNPs detachment from the liposomes in biological environments (e.g., serum, where proteins could displace gold).^31^ In our system, covalent anchoring of AuNPs was enabled by incorporating a commercially available thiol-functionalized lipid, 1,2-dipalmitoyl-*sn*-glycero-3-phosphothioethanol (16:0 Ptd Thioethanol, **DPSH**). To the best of our knowledge, the direct covalent conjugation of AuNPs to liposomes via this specific thiol-containing phospholipid has not been previously reported.

**• Electrostatic attraction** between AuNPs and liposomes increases coating efficiency because, in the reaction mixture, AuNPs accumulate around the liposomes instead of dispersing freely in the solution. As AuNPs accumulate around the liposomes, they simultaneously come into close proximity to thiol groups, facilitating covalent bond formation within the nanosystems. Efficient initial association between AuNPs and liposomes was facilitated by the incorporation of the cationic lipid 1,2-dioleoyl-3-trimethylammonium-propane (**DOTAP**), which provided a positive surface charge, promoting electrostatic attraction toward citrate-stabilized AuNPs.

Direct covalent attachment of gold to the thiol group present in the commercially available lipid eliminates the need to add additional building blocks to the lipid mixture or to further functionalize prepared liposomes. Examples of such modifications tested by other research teams include the functionalization of liposomes using thiol-terminated PEGs^23^ or incorporating 1-undecanethiol into a bilayer.^25^ Other research teams have opted for additional modification of citrate-stabilized AuNPs, for example, by coating them with cysteamine^32^ or halides.^33^ Our approach simplifies the process and allows for precise control over liposome properties by eliminating the need to introduce elements other than lipids. Simultaneously, incorporation of the cationic lipid DOTAP enables electrostatic preorganization of negatively charged AuNPs around the liposomes, increasing local nanoparticle concentration at the membrane surface and facilitating subsequent Au–S bond formation. Liposome building blocks, along with the simplified concept behind our approach, are presented in Figure 1. Second, we introduce practical quality control parameters that enable rapid confirmation of the AuNPs’ attachment to the liposome surface. These adjustments enabled the development of an efficient one-pot reaction that is readily scalable by avoiding multistep surface modifications and intermediate purification steps, which are often difficult to control and translate. A reaction limited to a single step offers several advantages: it reduces chemical waste, shortens overall synthesis time, and streamlines laboratory procedures by eliminating the need for multiple purification or transfer steps—an approach particularly attractive for basic research and industrial settings.^34^

**Figure 1:**
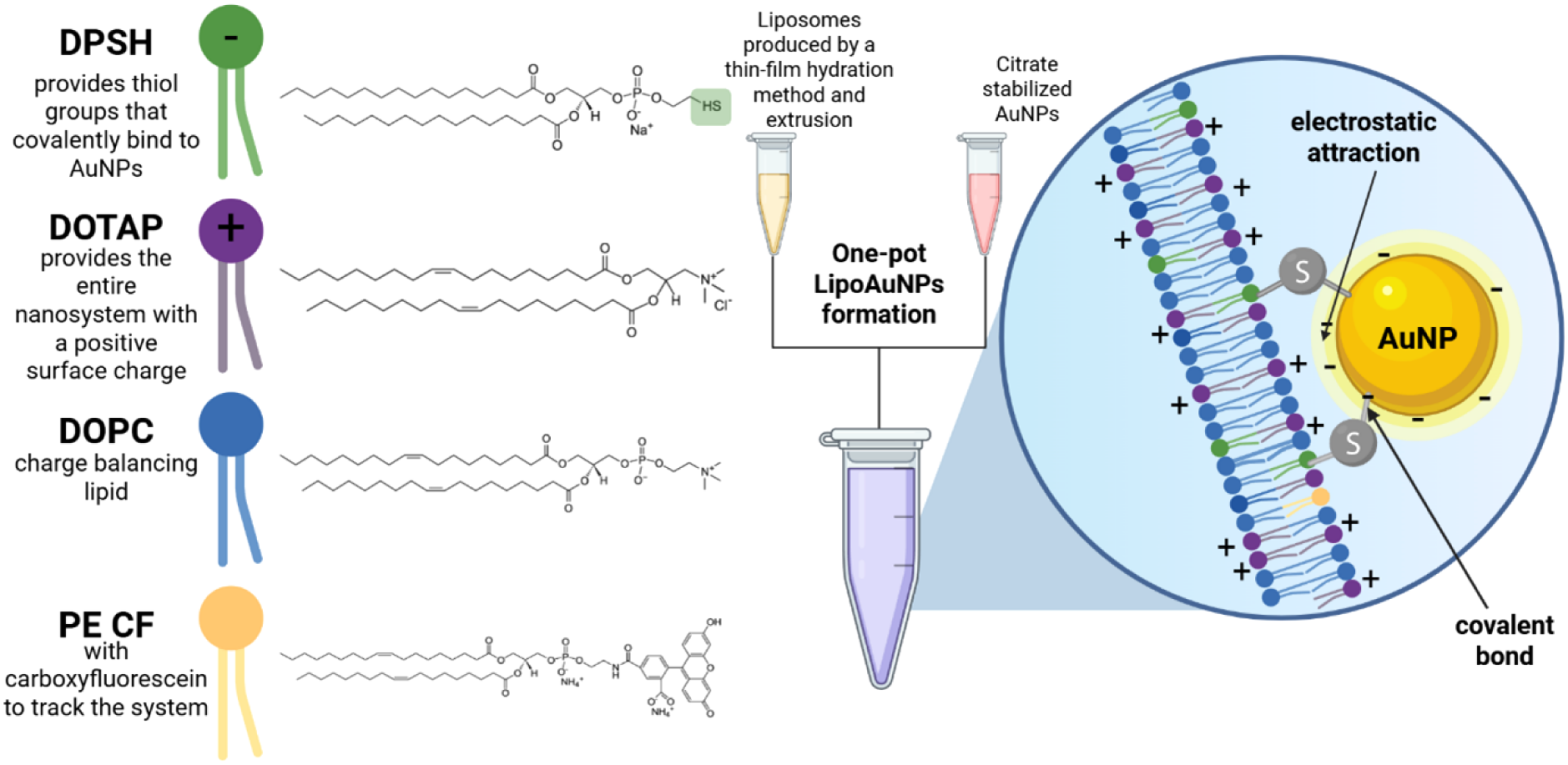
Scheme showing the chemical structures of phospholipids used to formulate liposomes subjected to surface modification with AuNPs (left). Visualization of the method’s simplicity based on a one-pot reaction (right).

Equally important is that the applicability of the obtained LipoAuNPs was confirmed by extensive biological studies. Certain aspects of biological studies (e.g., the effect of AuNP attachment to the liposome surface on its cellular uptake and trafficking) constitute new knowledge in the field of AuNP-based liposome research. While numerous studies have explored methods of synthesizing Au-modified liposomes via different *ex situ* approaches,^23,24,35^ detailed investigations into their cellular interactions remain limited. It is worth noting that LipoAuNPs represent a highly diverse class of nanoconstructs. Some reports focus on AuNPs encapsulated within the liposomal cargo, others incorporate them into the liposomal bilayer, while some, such as Dichello et al.,^36^ position them between multiple layers in multilamellar layersomes. Even in *ex situ* approaches, the biological activity of these nanoconstructs can be strongly influenced by the choice of liposomal lipid composition and the type of AuNPs employed. In this study, we investigated how the attachment of AuNPs to the liposome surface influences their uptake by cancer cells and their intracellular trafficking using flow cytometry and fluorescence confocal microscopy. Gaining insight into the impact of AuNPs on cellular fate is essential for advancing the use of this carrier system in cancer therapy.

## 2 Materials and Methods

### 2.1 Chemicals and Materials

#### 2.1.1 Electrochemical Studies

Five millimolar solution of bis­(triphenylphosphoranylidene)­ammonium tetrakis­(4-chlorophenyl) (BTPPATPBCl) dissolved in 1,2-dichloroethane (1,2-DCE, Sigma-Aldrich, 99%) was used as the organic phase background electrolyte. It was prepared by a simple metathesis reaction from solutions of potassium tetrakis­(4-chlorophenyl) (KTPBCl, Sigma-Aldrich, 98%) dissolved in methanol (MeOH, POCh, pure) and bis­(triphenylphosphoranylidene) ammonium chloride (BTPPACl, Sigma-Aldrich, 97%) dissolved in a methanol:water 2:1 (v:v) mixture. The precipitate was filtered and cleaned with a water:acetone (POCh, pure) 2:1 (v:v) mixture and further recrystallized from pure acetone. The aqueous solution of 10 mM sodium chloride (NaCl, POCh, 99%) and BTPPACl was used as the organic phase reference electrode supporting phase.

#### 2.1.2 Synthesis of Gold Nanoparticles

Gold­(III) chloride trihydrate was purchased from Sigma-Aldrich. Trisodium citrate dihydrate was obtained from Chempur.

#### 2.1.3 Liposome Formation

The following lipids were obtained from Avanti Polar Lipids: 1,2-dioleoyl-*sn*-glycero-3-phosphocholine (DOPC), 1,2-dipalmitoyl-*sn*-glycero-3-phosphothioethanol (16:0 Ptd Thioethanol, DPSH), 1,2-dioleoyl-3-trimethylammonium-propane (DOTAP), and 1,2-dioleoyl-*sn*-glycero-3-phosphoethanolamine-N-carboxyfluorescein (18:1 PE CF). The purchased lipids were in the form of a powder or a ready-to-use stock solution in chloroform. In the case of the powder, the predefined amount of lipids was dissolved in chloroform for further use.

#### 2.1.4 Cellular Studies

3-(4,5-Dimethylthiazol-2-yl)-2,5-diphenyltetrazolium bromide (MTT) and 4′,6-diamidine-2′-phenylindole dihydrochloride (DAPI) ready-made solutions were purchased from Sigma-Aldrich. CellMask Plasma Membrane Stains and LysoTracker Deep Red were obtained from Invitrogen. Trypan blue was purchased from Gibco.

Two immortalized carcinoma cell lines were chosen for *in vitro* research: HeLa and HepG2. Both cell lines were purchased from ATCC (USA). The cell culture medium (DMEM with GlutaMAX) was obtained from Gibco and further enriched with FBS (10% v/v) purchased from BioWest, and a mixture of penicillin and streptomycin (1% v/v) from Gibco. Flasks were obtained from Nunc (Germany).

### 2.2 Liposomes Formation

Two phospholipid compositions were used to prepare the liposomes. In both formulations (hereafter referred to as “DPSH Liposomes” and “DOTAP&DPSH Liposomes”), 10% of the lipid mass consisted of DPSH, with thiol functionalities located within the polar head groups, and 2% consisted of 18:1 PE CF, with carboxyfluorescein attached to the phosphate group through an amide linkage. The thiol groups enable strong covalent bonding with AuNPs, while carboxyfluorescein was added to enable the tracking of liposomes *in vitro* by flow cytometry and fluorescence confocal microscopy. The DOTAP&DPSH Liposomes formulation contained 10% DPSH and 25% DOTAP by mass, providing the liposomes with a positive surface charge and thiol group terminations. In both variants, the remaining lipid mass was completed with zwitterionic DOPC, which acts as a charge-balancing lipid.

Before liposome preparation, all lipids were dissolved separately in chloroform or purchased as a chloroform solution. Next, the appropriate aliquots of the lipid solution were transferred to a glass vial, where chloroform was carefully evaporated under a gentle stream of argon. To eliminate any remaining chloroform, samples were placed in a desiccator and kept under vacuum for 30 min. After this, a thin lipid film was visible at the bottom of the vessel. During the following step, x mL of 10 mM Tris buffer was added to the glass vial with the lipid film (x : volume was selected based on the desired final concentration needed, i.e., 3 or 9 mg mL^–1^). Next, samples were alternately sonicated in an ultrasonic bath and vortexed in order to suspend the entire lipid film in the aqueous phase. (At this stage, the formation of multilamellar vesicles is expected.) The unilamellar liposomes were obtained with the extrusion method using an extruder by Avanti Polar Lipids. In total, the liposome solution was pressed 21 times through the Whatman polycarbonate Nuclepore filter discs with a 0.1 μm pore size placed between two Whatman Polyester Drain Discs. The glass syringes used during the extrusion process had a volume of 1 mL.

### 2.3 AuNPs Synthesis

AuNPs were synthesized according to the modified Turkevich method.^37,38^ Briefly, a 1 mM solution of tetrachloroauric acid (III) trihydrate was placed on a magnetic stirrer and heated to 100 °C. Then, a 30 mM solution of trisodium citrate dihydrate was added to obtain a citrate/Au ratio of 3:1. The mixture was continuously stirred for around 4 min (while experiencing the gradual color change from yellow, through colorless, black, to dark purple). After this time, the obtained solution of AuNPs was cooled and stored at 4 °C.

### 2.4 LipoAuNPs Conjugate Formation

#### 2.4.1 Primary Method

LipoAuNPs were obtained by the addition of liposomes into the AuNPs solution, prepared as described above. Depending on the proportion of AuNPs to liposomes, different mass concentrations of lipids were used (9 mg mL^–1^ for DPSH liposomes and 3 mg mL^–1^ for DOTAP&DPSH liposomes). The proportion of liposomes to AuNPs was selected in such a way as to attach as many AuNPs as possible to the liposome without the formation of aggregates. In each case, 220 μL of AuNPs were used per 100 μL of liposomes. The method allowed for scale-up in order to obtain the desired volume of AuNPs. To form conjugates, the AuNPs solution was placed in Eppendorf tubes on a stirrer (350 rpm, room temperature), and then liposomes were added. The resulting mixture was left on the stirrer for 2 h to ensure complete adsorption of AuNPs to the liposome surface, driven by electrostatic interaction and the formation of covalent bonds between the thiol group of DPSH lipid and the metallic NPs surface.

#### 2.4.2 Modified Method

In the case of DPSH liposomes, additional steps were introduced to ensure the attachment of AuNPs to the liposome surface. Before the addition of liposomes to AuNPs, the liposome solution was acidified with 1 M HCl to the point of zeta potential charge reversal from negative to positive. Next, the obtained solution was used to modify DPSH liposomes according to the procedure described in section 2.4.1, but in this case, liposomes were added in 50 μL aliquots every 3 min. Once the final mixture was obtained, LipoAuNPs were dialyzed. To do so, the obtained solution was loaded into a sac made of SnakeSkin Dialysis Tubing from Thermo Scientific. The sac was carefully placed into a flask filled with 1 L of a 10 mM Tris buffer solution. The flask was placed on a magnetic stirrer located in a walk-in refrigerator and left overnight (300 rpm and 6 °C). The effectiveness of the dialysis was assessed by pH measurements.

### 2.5 Characterizations

#### 2.5.1 Hydrodynamic Diameter and Zeta Potential Measurements

The hydrodynamic diameter of the nanosystems was measured by using the dynamic light scattering (DLS) method. For this purpose, the Zetasizer Nano ZS and Malvern software were used. Each sample was characterized by three measurements, consisting of the number of runs automatically set based on polydispersity. Before the measurement, samples were diluted to reach a 0.1 mg mL^–1^ lipid concentration. Zeta (ζ) potential measurements were also performed by using Zetasizer Nano ZS and Malvern software. For the measurement, samples were diluted in Tris buffer at pH 7 and 10 to 0.5 mg mL^–1^ lipid concentration.

#### 2.5.2 Fluorescence and Absorbance

Fluorescence measurements of liposomes tagged with lipids modified with carboxyfluorescein were conducted by using a PerkinElmer LS55 spectrofluorometer. To ensure accurate fluorescence readings, samples were diluted in 10 mM Tris buffer to a lipid concentration of 0.125 mg mL^–1^ or lower. Absorbance was measured by using a Jasco V-650 UV–vis spectrophotometer. For reliable absorbance data, sample dilutions were adjusted to maintain absorbance values below 1.

#### 2.5.3 Transmission Electron Microscopy

The LipoAuNPs complexes were visualized using a JEOL-1010 (JEOL, Tokyo, Japan) transmission electron microscope. Samples, without additional dilution, were placed on 200-mesh copper grids with a carbon surface (Ted Pella, Inc., Redding, CA, USA). Samples were stained with 2% uranyl acetate for 2 min, washed with deionized water, and dried at room temperature. Images were taken at a magnification of 100,000×. The developed films were scanned using a Perfection V700 PHOTO scanner (Epson).

### 2.6 Lipid Monolayers–AuNPs Interaction

AC voltammetry (ACV) measurements were performed in a biphasic system (interface between two immiscible electrolyte solutions: ITIES) to follow the adsorption of lipids at the liquid–liquid interface and their further interaction with the AuNPs. A four-electrode configuration with a dedicated glass cell was used in this respect. The electrochemical configuration is schematically shown in Figure S1A. Two Pt electrodes served as the counter electrodes, each placed in the aqueous and organic phases. The electric contact of the organic phase Pt (counter) electrode was additionally covered with a glass tube to avoid any short circuits with the aqueous phase. Both reference electrodes were made of Ag wire covered with AgCl (oxidation of Ag in saturated FeCl_3_ solution). The aqueous phase reference electrode was placed into the Luggin capillary of the upper cell compartment filled with 10 mM NaCl, whereas the organic phase reference electrode was placed in the bottom Luggin capillary, additionally filled with the aqueous supporting solution of 10 mM NaCl and 10 mM BTPPACl. Since the concentration of Cl^–^ anion in the Ag/AgCl wires contacting the phases did not change during the measurement, the potential of both (and between) electrodes remained constant. During the measurements, lipids were added directly to the organic phase (see Figure S1B) or drop-cast over the organic phase surface before the aqueous phase placement (see Figure S1C). The biphasic system was polarized with the help of an external power source – Autolab 302n potentiostat-galvanostat. AC voltammograms were recorded with NOVA 2.1 software after application of the following parameters: step: 5 mV; modulation amplitude: 25 mV vs RMS; modulation time: 0.2 s; frequency: 6 Hz. Scheme 1 shows the electrochemical cell configuration.

**Scheme 1:**
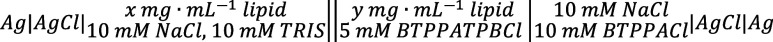
Representation of the Electrochemical Cell Used to Study Interactions between Lipid Monolayers and AuNPs

### 2.7 Cell Culture

Cells were cultured in T-75 culture flasks in a humidified atmosphere containing 5.0% CO_2_ at 37 °C and were passaged every 2 or 3 days. Cells were harvested and used in experiments after obtaining 80–90% confluence. The number of viable cells was determined by the trypan blue exclusion assay with the use of an automated cell counter (EVE NanoEnTek).

### 2.8 Liposome Uptake

#### 2.8.1 Measurement by Flow Cytometry

*In vitro* uptake studies were carried out by using flow cytometry. Cells were seeded on 12-well plates (HeLa: 1.0 × 10^5^ per well, HepG2: 1.5 × 10^5^ per well) in 1 mL of appropriate medium and were incubated for 24 h in a humidified atmosphere containing 5.0% CO_2_ at 37 °C in order to allow cells to attach to the plates. Then, liposomes were added so that their final mass concentration on the plate was 10 μg·mL^–1^ or 40 μg·mL^–1^. Liposomes were added at time intervals, allowing them to incubate with the cells for 2, 4, 8, and 24 h. After incubation with liposomes, the cell medium was collected from the plates, and the wells were washed with phosphate-buffered saline (PBS). Then, the cells were detached by using 150 μL of standard trypsin, and 350 μL of medium was added to the detached cells. Next, the cell suspension was directly transferred to flow cytometry tubes. The cells were measured on a cytometer immediately after collection without additional fixation. To estimate cellular uptake, fluorescence was measured using flow cytometry (BD FACSymphony A1 Cell Analyzer). For excitation, the 488 nm laser line was used, and fluorescence was detected in the FITC channel (530 ± 15 nm). In each experiment, 10,000 events were counted. As a control, untreated cells were measured, and their autofluorescence intensity was calculated and subtracted from each experimental result. The findings are presented as the percentage of cells in the population that internalized carboxyfluorescein attached to the liposomal membrane or as mean fluorescence. A figure showing exemplary results and gating is included in the Electronic Supporting Information (Figure S2).

#### 2.8.2 Measurement by Flow Cytometry with Trypan Blue Staining

To obtain results of fluorescence originating exclusively from liposomes internalized inside the cells, and not those adsorbed to the cell surface, trypan blue (a quencher of carboxyfluorescein fluorescence) was added.^39^ Additionally, trypan blue is actively removed from the inside of living cells; therefore, it only quenches external fluorescence. For such a measurement, all steps were carried out as above; however, just before the measurement, 50 μL of trypan blue was added to a 500 μL suspension of cells. The volume of trypan blue was determined experimentally by using a PerkinElmer LS55 spectrofluorometer. The fluorescence quenching curve for the tested concentration of liposomes by trypan blue is included in the Supporting Information (Figure S3).

#### 2.8.3 Visualization by Confocal Microscopy

Cells were seeded onto IBIDI μ-Slide 18 Well Glass Bottom plates (HeLa: 1.5 × 10^3^ cells·well^–1^; HepG2: 3.0 × 10^3^ cells·well^–1^) in 100 μL of the appropriate culture medium. Following seeding, cells were incubated under standard culture conditions (37 °C, 5.0% CO_2_) for 48 h to ensure complete adhesion and the development of characteristic cellular morphology, which is particularly important given the specific properties of the glass-bottom plates. After the 48 h incubation, liposomes were added to the wells at a final concentration of 10 μg·mL^–1^. The cells were subsequently incubated with the liposomes for 4 h. Following incubation, the culture medium was carefully aspirated, and the cells were subjected to staining procedures for confocal microscopy.

To evaluate the colocalization of liposomes with acidic compartments such as late endosomes and lysosomes, LysoTracker Deep Red was employed. A working solution of LysoTracker (75 nM) was prepared in the culture medium, and 100 μL was added to each well, followed by a 45-min incubation under standard conditions. After staining, cells were washed three times with DPBS and fixed with 4% formaldehyde for 15 min at room temperature. The cells were then washed with DPBS, followed by the addition of 100 μL of DPBS containing DAPI (1 μg·mL^–1^) to each well for nuclear counterstaining. Finally, the plates were protected from light by wrapping in aluminum foil and stored at 4 °C until confocal imaging.

Microscopic imaging was performed using the TCS SP8 confocal laser scanning microscope (Leica Microsystems, Germany), equipped with a 63×/1.40 oil immersion objective (HC PL APO CS2, Leica Microsystems, Germany). Samples were visualized using the following excitation and emission wavelength settings: DAPI: 405 nm excitation/430–470 nm emission; carboxyfluorescein: 488 nm excitation/520–540 nm emission; LysoTracker Deep Red and CellMask True Red: 575 nm excitation/660–670 nm emission. Fluorescence intensity and colocalization analyses were presented in arbitrary units (a.u.) using the Leica Application Suite X (LAS X, Leica Microsystems, Germany).

#### 2.8.4 EDX Analysis

To gain additional insight into the nature of interactions between liposomes containing the thiol-functionalized lipid (DPSH) and gold surfaces, we used energy-dispersive X-ray spectroscopy (EDX).

Briefly, a 20 nm thick gold layer was deposited onto copper wires using a Leica EM ACE200 low-vacuum coater. The gold-coated wires were then bent to form a mesh, further incubated with two types of liposomal suspensions under gentle stirring for 2 h: liposomes composed exclusively of DOPC and liposomes composed of DOPC supplemented with 10 wt % of DPSH. After incubation, the substrates were rinsed thoroughly three times with Tris buffer to eliminate nonadsorbed liposomes or lipid-based structures. After drying the sample surface under a stream of argon, EDX analysis was performed (Phenom SEM-EDX analyzer; 20 kV was used as the acceleration voltage). A control experiment with Au-coated glass was also performed to prove the absence of any P- or S-based contaminations.

### 2.9 Cytotoxicity Assessment

#### 2.9.1 MTT Assay

Cells were seeded in flat-bottom 96-well plates at a density of 1.0 × 10^4^ cells·well^–1^ for the HeLa cell line and 1.5 × 10^4^ cells·well^–1^ for HepG2 in 100 μL of an appropriate medium. After seeding, plates were incubated for 24 h in a humidified atmosphere containing 5.0% CO_2_ at 37 °C to allow cells to attach to the plates. After approximately 24 h, 10 μL of liposomes diluted in Tris buffer were added to the cells in a concentration range from 5 to 200 μg·mL^–1^. Ten microliters of Tris buffer was added as a control (its lack of cytotoxicity has been previously verified—data not shown). Cells were incubated with the liposomes for another 24 h in a 37 °C humidified atmosphere containing 5.0% CO_2_. After the incubation, cells were washed with PBS. Next, 50 μL of a 0.5 mg·mL^–1^ solution of MTT in PBS was added to each well, and cells were further incubated under standard culture conditions for 3 h. After incubation, the residual MTT solution was removed, and the obtained formazan precipitate was dissolved in DMSO (100 μL·well^–1^). The conversion of tetrazolium salt (MTT) to a colored formazan by mitochondrial and cytosolic dehydrogenases is a marker of cell viability. To determine the amount of dissolved formazan, the color intensity of the sample was measured by a Synergy HTX reader. The absorbance of light at 570 nm was measured, from which the absorbance at 720 nm (background reference wavelength) was subtracted. The percentage of live cells was then calculated, taking the absorbance of cells treated only with buffer as 100%.

#### 2.9.2 Real-Time Monitoring of Cell Viability Based on Cellular Impedance

Real-time impedance measurements were performed by using xCELLigence hardware, software, and 16-well plates. The first stage of the experiment was to add 50 μL of cell medium to each well of a 16-well plate and measure the impedance value. Then, 100 μL of cell suspension was added so that their density on the plate was 1.0 × 10^4^ cells·well^–1^ in the case of the HeLa line and 1.5 × 10^4^ cells·well^–1^ in the case of the HepG2 line. The cells were incubated for 24 h, after which the appropriate liposome variant was added to obtain final concentrations of 200 μg·mL^–1^. Cells with the compounds were incubated for 24 h, and each liposome variant was monitored in four wells. The impedance measurement was performed automatically every 15 min, and its value was given as the Cell Index (CI) parameter. After the experiment, the CI values for individual concentrations were averaged.

### 2.10 Statistical Analysis

To evaluate differences in cellular uptake among various liposome formulations over time, a two-way ANOVA was performed using GraphPad Prism (version 10.4.1). The analysis included two factors: liposome formulation (between-subject factor) and incubation time (within-subject factor). A full model was applied, testing for the main effects of both factors, as well as their interaction. For post hoc analysis, Tukey’s multiple comparisons test was applied to compare mean fluorescence values between all liposome groups at each individual time point.

To assess whether liposome type and lipid concentration had a significant effect on cell viability and whether there was an interaction between these two factors, a two-way ANOVA was performed. To assess the effect of liposome charge on cytotoxicity and the effect of AuNPs attachment, multiple comparisons were performed within the two-way ANOVA framework with Šídák’s multiple comparisons test for both cell lines. Additionally, mean cell viability results for each concentration were compared to the control sample by Dunnett’s multiple comparisons test, with a single pooled variance, within the two-way ANOVA framework. Statistical significance was set at *p* < 0.05.

## 3 Results and Discussion

### 3.1 Liposomes and LipoAuNPs Characterization

The primary objective of this study was to obtain stable LipoAuNPs conjugates, with the anticipated outcome being an increase in the hydrodynamic diameter of liposomes due to the attachment of AuNPs to their surface. A simple mixing approach of DPSH liposomes with citrate-capped AuNPs was attempted first. However, contrary to expectations, no stable conjugates were formed using this method. Instead, the hydrodynamic diameter of the resulting mixture was found to be slightly smaller (94.30 ± 0.72 nm) than that of bare liposomes (111.70 ± 0.92). Furthermore, dynamic light scattering analysis revealed two distinct particle populations (Figure S4B): a main peak of approximately 136 nm, corresponding to the liposome fraction, and a second smaller peak near 31 nm, representing unbound AuNPs. Additionally, an increased PDI value of 0.277 for the investigated mixture further confirms a higher level of heterogeneity compared to pure DPSH liposomes. The unsuccessful conjugation via the simple mixing approach was attributed to electrostatic repulsion between negatively charged citrate-stabilized AuNPs and negatively charged DPSH liposomes containing phosphate groups. The electrostatic hindrance likely prevented the formation of stable Au–S bonds, which are essential for effective conjugation.

The electrostatic repulsion of DPSH liposomes and AuNPs was successfully overcome by the initial acidification of the DPSH liposomes suspension with 1 M HCl. The charge reversal (from negative to positive) of the liposome surface, resulting from the protonation of the phosphate group of DPSH and DOPC lipids, eliminated the electrostatic repulsion barrier and allowed the AuNPs to adsorb to the liposomal surface. Consequently, after the addition of acidified DPSH liposomes to the AuNP solution, their average size increased from around 111 nm to around 155 nm. Given that the average diameter of the AuNPs is around 12 nm, the increase in size, equal to 44 nm, holds the expected order of magnitude. A small discrepancy is related to the experimental approach employed, which one should keep in mind when analyzing these data sets. The reliable average AuNP diameter was determined based on TEM microscopy imaging (see micrographs showing AuNPs with their diameters presented in Figure S6 in the Supporting Information) while the examination of conjugates was only possible with DLS-based measurements. Low PDI values (below 0.3) of conjugates indicate the monodispersity of the samples and qualify the obtained LipoAuNPs for further studies. However, the acidification of liposomes with hydrochloric acid resulted in the necessity of performing another step—dialysis, as (i) the medium with low pH cannot be applied for cellular uptake and trafficking studies due to the cytotoxic effect of HCl; (ii) the fluorescence of carboxyfluorescein is reduced in an acidic environment,^40^ and finally, (iii) the addition of the LipoAuNPs stored in the acidic solution to the buffer at physiological pH may affect their physicochemical properties. Interestingly, as shown in Table 1 (see the column “After dialysis”), neither the hydrodynamic size (obtained values fall within the error margin) nor the PDI (<0.3) was affected by the dialysis process and by restoring the pH to 10. All these findings suggest that, despite the initial electrostatic repulsion, acidification-induced charge reversal enables the stable attachment of AuNPs to the liposome surface.

**Table 1:** Comparison of Hydrodynamic Diameters along with PDI of the Tested LipoAuNPs Nanosystems^a^

Parameter	DPSH Liposomes	DPSH Liposomes + AuNPs	DPSH Liposomes + HCl + AuNPs	After dialysis: DPSH Liposomes + AuNPs
**Zeta average size [nm]**	111.70 ± 0.92	94.30 ± 0.72 (no conjugates’ formation)	155.00 ± 4.12	155.50 ± 3.88
**PDI**	0.087 ± 0.016	0.277 ± 0.007	0.171 ± 0.018	0.227 ± 0.014
**Size of the main peak [nm]**	120.00 ± 2.10	136.40 ± 3.86	181.50 ± 6.10	185.20 ± 8.70

a The Presented Results are the Average of 3 Measurements ± Standard Deviation.

It is worth noting that our results confirm that the increase in the hydrodynamic diameter of acidified DPSH liposomes after incubation with AuNPs is not related to AuNP aggregation induced by the acidic environment itself. In a control experiment, AuNPs were treated with the same amount of HCl under identical conditions but in the absence of liposomes. Upon HCl addition, the AuNP dispersion rapidly changed color from red to purple and, subsequently, to gray, and visible aggregates appeared within minutes. This behavior was accompanied by a dramatic increase in hydrodynamic diameter measured by DLS, confirming extensive nanoparticle aggregation resulting from the loss of surface charge stabilization (results are shown in Figure S7). Under strongly acidic conditions (pH 2.3), citrate ligands are likely detached from the AuNP surface. In contrast, in the presence of DPSH liposomes, AuNPs are rapidly stabilized, which we attribute to thiol-mediated Au–S interactions with functional groups exposed at the liposome surface, preventing uncontrolled aggregation. Acidification, therefore, primarily serves to eliminate electrostatic repulsion rather than to induce electrostatic attraction between AuNPs and liposomes.

Even though the LipoAuNPs conjugate formation was achieved for DPSH liposomes, the need for an additional dialysis step is considered a drawback from an applicability point of view. Limitations related to potential scale-up and batch-to-batch variations were a motivation to pursue other conjugate formation protocols, ultimately leading to a very straightforward single-pot methodology meeting the standards of the rational design of carriers.^41^

To enable the initial adsorption of negatively charged AuNPs to liposomes, the positively charged lipid – DOTAP, terminated with a quaternary ammonium group, was added to the initial lipid formulation. The use of DOTAP was not accidental, as other research teams had already proven its utility in AuNPs placement on the liposomal surface.^26,28^ We, in turn, propose an innovative modification. Therefore, after the initial electrostatic attachment to the positively charged polar headgroup of DOTAP, a strong covalent bond was formed between the thiol groups of the DPSH lipids and the AuNPs. Such a modification is expected to provide stable conjugates even when exposed to local pH gradients.

The measurement of zeta potential allowed us to conclude that the addition of 25% DOTAP (by mass) to the lipid mixture increases zeta potential from around −36 mV to around 38 mV at pH 7.0—see Figure 2A. Bare DOTAP&DPSH liposomes are characterized by slightly higher hydrodynamic diameter and PDI than DPSH liposomes (e.g., DOTAP&DPSH liposomes are ∼130 nm and DPSH liposomes are ∼110 nm). Crucially, the direct addition of DOTAP&DPSH liposomes to the AuNPs solution results in a shift in the mean hydrodynamic diameter from ∼130 nm to ∼150 nm. This increase in diameter by approximately 20 nm corresponds to the diameter of nearly two AuNPs (as measured by TEM micrograph analysis). A comprehensive overview of the DOTAP&DPSH liposome size affected by the AuNPs modification process is provided in Table 2 and Figure S5. The lower increase in hydrodynamic diameter in the case of DOTAP&DPSH liposomes than in DPSH liposomes may be related to a presumed change in lipid packing (especially within the polar headgroup region) within the liposomal bilayer, affected by charge screening observed as a drop in ζ-potential after AuNPs adsorption—see Figure 2A showing the drop in ζ-potential from 38 mV to 15 mV (at pH 7.0). In other words, charge neutralization is expected to reduce the distance between evenly charged lipids located in the lipid bilayer, thus causing a drop in liposome diameter. This is in line with many reports focused on the study of lipid organization in the lipid bilayer affected by acid–base equilibria, adsorption of inorganic ions or NPs, and the resulting effect on ζ-potential,^42^ electric properties of the bilayer^43,44^ its interfacial tension.^45^ Other explanations are also possible, as the effect of AuNPs on liposome size downscaling was also evidenced by Tehrani et al., who attributed the change to “complex reorganization of the lipid bilayer” and the formation of multilamellar vesicles, but only for particular lipidic compositions.^46^ Even though this is an interesting observation, its further investigation was beyond the scope of this work.

**Table 2:** Comparison of Hydrodynamic Diameters of Tested DOTAP&DPSH Liposomes before and after Modification with AuNPs^a^

Parameter	DOTAP&DPSH Liposomes	DOTAP&DPSH Liposomes + AuNPs
**Zeta average size [nm]**	133.6 ± 3.4	150.5 ± 1.0
**PDI**	0.194 ± 0.015	0.160 ± 0.006
**Size of the main peak [nm]**	145.2 ± 6.2	179.9 ± 2.1

a The Presented Results are the Average of 3 Measurements ± Standard Deviation.

**Figure 2:**
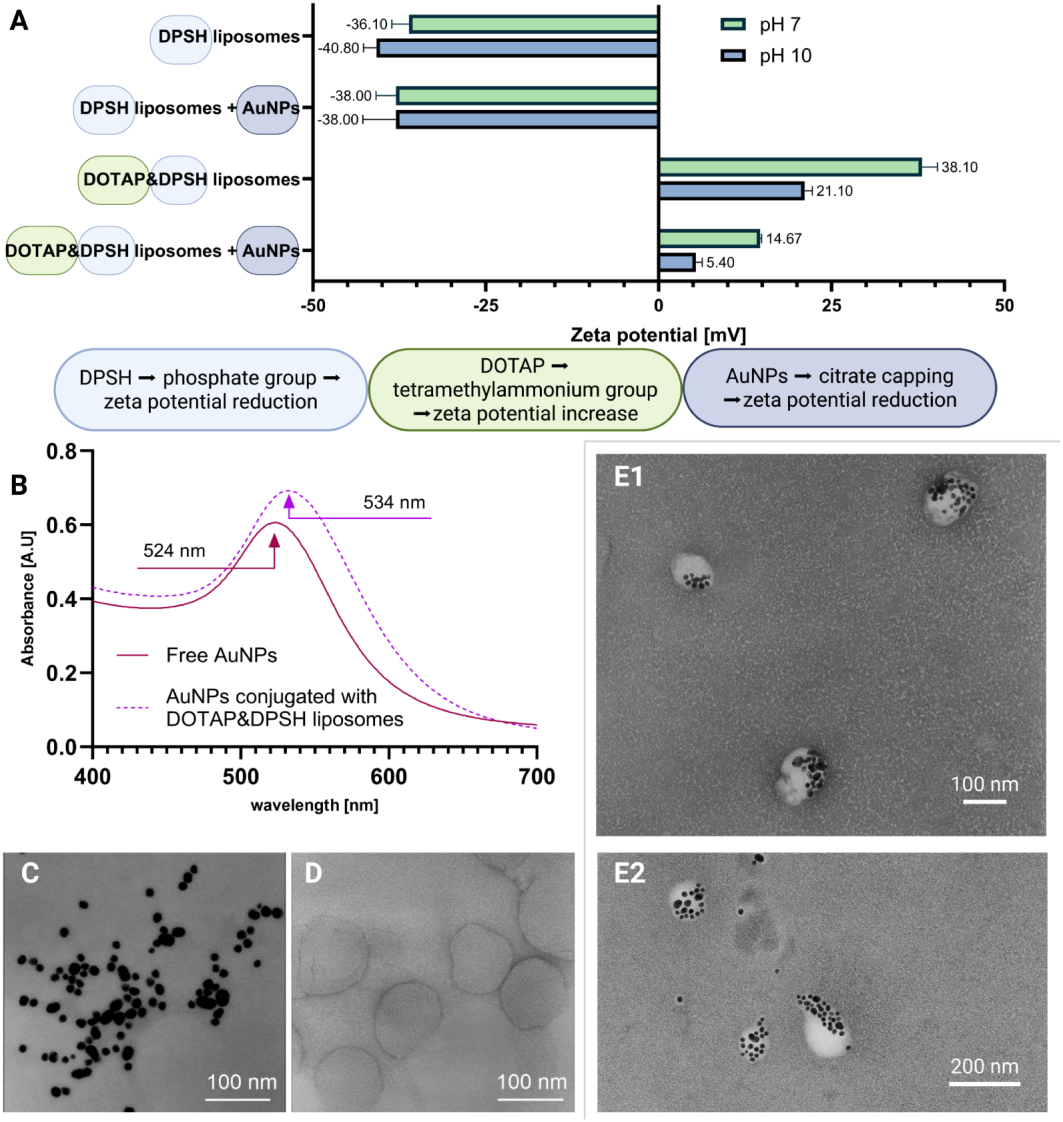
(A) Comparison of the zeta potentials of the studied nanosystems. Measurements were performed at a mass concentration of lipids of 0.5 mg mL^‑1^; liposomes were dissolved in Tris buffers at pH 7 and 10. The presented results are the average of 3 measurements ± standard deviation. (B) UV–Vis spectra recorded for AuNPs (red solid line curve) and DOTAP&DPSH liposomes modified with AuNPs after bare DOTAP&DPSH liposomes absorption subtraction (violet dashed line curve). (C) Transmission electron microscopy micrograph of citrate-stabilized AuNPs. (D) Transmission electron microscopy micrograph of DOTAP&DPSH Liposomes. (E1,2) Transmission electron microscopy micrographs of DOTAP&DPSH liposomes after modification with AuNPs, captured at two different magnifications.

This effect was not observed for DPSH liposomes, as here the ζ-potential before and after AuNPs addition remains nearly unaffected (Figure 2A). Also, we have studied the responsiveness of the ζ-potential to a slight increase in the solution pH (from 7.0 to 10.0). The change in the studied parameter was especially visible for DOTAP&DPSH liposomes and attributed to the hydrolysis of the phosphate in the polar headgroup of DPSH—see Figure 2A. All these results indicate that the acid–base equilibria can be used to fine-tune the surface charge of liposomes during a modification process.

Successful surface attachment of AuNPs to DOTAP&DPSH liposomes was also manifested in the shift of the absorbance peak of AuNPs. Figure 2B shows the UV–vis spectra recorded for the AuNPs solution and AuNPs conjugates with DOTAP&DPSH liposomes. The bathochromic shift of the *λ*_*max*_ is typical for the AuNP aggregation process and may be used as an indication of the metallic particles’ adsorption to the liposome surface,^47^ where plasmon resonance coupling is observed. This observation further supports the claim that AuNPs were successfully placed on the studied liposome surface.

To further verify the formation and stability of the DOTAP&DPSH liposomes modified with AuNPs, transmission electron microscopy (TEM) analysis was conducted. The size of the AuNPs, as obtained with TEM (see Figure 2C), was subjected to statistical analysis provided in Figure S5. A good correlation (taking into account the sample preparation procedure) was also observed for the size of DOTAP&DPSH liposomes that are shown in Figure 2D—diameters of ∼100 nm were obtained. Finally, the resulting images clearly demonstrated the presence of well-defined nanosystems with distinct AuNPs visibly accumulated around the liposomes (Figure 2E). While the images confirmed the successful attachment of AuNPs, it was evident that the distribution was not entirely uniform across all liposomes. It is important to note that TEM sample preparation involves drying, blotting, and storing under vacuum, which influences the native structure and spatial arrangement of the NPs and liposomes, and should be considered only as an additional inspection method.^48^

LipoAuNPs characterization allowed us to define two quality control tests confirming the attachment of AuNPs to the liposome surface, which can easily be implemented into routine LipoAuNPs preparation protocols. Naturally, the first quality control factor is an increase in the mean hydrodynamic diameter of liposomes after mixing them with AuNPs, corresponding to the order of magnitude of a diameter of approximately two AuNPs (one should keep in mind that a size distribution of a population of AuNPs with a mean diameter of ∼20 nm will cover 2 orders of magnitude). This increase should not be accompanied by a significant increase in PDI, as this could indicate an uneven distribution of AuNPs in the sample or the formation of aggregates consisting of several liposomes. Note that the increase in hydrodynamic diameter specifically indicates that the AuNPs are located on the outer surface of the liposomes, rather than being incorporated into the bilayer or aggregating within intraliposomal membrane cavities, as described by Contini et al.^49^ Another method developed to quickly confirm the adsorption of AuNPs to the liposome surface is to spectrophotometrically confirm the shift of the absorbance peak of AuNPs upon attachment to liposomes. In our case, after adsorption, we could observe a 10 nm shift of the *λ*_*max*_.

To further understand the nature of liposome–gold interactions, we used EDX. Gold-coated copper substrates were incubated with liposomes of different compositions. This approach aims to assess whether liposomes containing a thiol-functionalized lipid may adsorb to the gold surface after extensive washing. In the case of liposomes containing DPSH, both sulfur and phosphorus signals were detected on the gold surface. Sulfur originates exclusively from thiol groups present in the DPSH lipid, while the phosphorus group is found in both DOPC and DPSH. The simultaneous presence of these elements provides strong evidence that DPSH-containing liposomes remain bound to the Au surface. In contrast, no phosphorus signal was detected after incubation of substrates covered with Au with DOPC-only liposomes, indicating that these liposomes were completely removed during the washing step—see figure S8 for details. This observation, although only qualitative, demonstrates that, in the absence of thiol functionalities, noncovalent interactions such as van der Waals forces are insufficient to maintain stable liposome attachment to Au. This experiment further supports our claim that thiol groups play a key role in the stabilization of LipoAuNPs formed from DPSH-based liposomes. However, as discussed in earlier sections, the formation of such covalent bonds requires prior elimination of electrostatic repulsion between negatively charged liposomes and AuNPs. In the case of LipoAuNPs derived from DOTAP&DPSH liposomes, strong electrostatic attraction is likely dominant at the initial adsorption stage and is subsequently reinforced by the formation of covalent Au–S bonds.

In terms of the biological activity of the nanosystem, it is important to highlight that the AuNP addition to DOTAP&DPSH liposomes results in a charge reduction of the entire DOTAP&DPSH + AuNPs conjugates, although the obtained LipoAuNPs remained positive (see Figure 2A). This provides a prognosis for effective transport across the cell membrane.^50^ The obtained LipoAuNPs also possess the advantage of storage stability (see Figure S9 in the electronic Supporting Information for details), and their formation is reproducible and controllable. In summary, the obtained DOTAP&DPSH + AuNPs conjugates have many advantages that make them promising nanosystems for biomedical applications, and therefore, it was decided to conduct thorough studies on their biological activity. While promising, it is important to note that the use of cationic liposomes may also be associated with increased cytotoxicity, reduced stability in the presence of serum, and decreased biocompatibility.^51^ Hence, the need for further biological studies is very high.

### 3.2 Liposomes–AuNPs Electrostatic Interaction Characterization

To further study the interaction between AuNPs and different lipid mixtures, we used interfacial electrochemistry. The electrified liquid–liquid interface (eLLI, or the interface between two immiscible electrolyte solutions, ITIES) was used to study the interaction between different lipids and ITIES, and between lipids adsorbed to the ITIES and AuNPs.

The biphasic platform, composed of the aqueous solution of the 10 mM NaCl and the organic phase (the solution of the organic phase background electrolyte dissolved in the 1,2-DCE) makes an intuitive model environment where the lipid monolayer can be formed (driven by spontaneous adsorption of amphiphiles to an asymmetric phase boundary defined by the polar and nonpolar solvent) and studied with a range of electrochemical techniques, ion transfer voltammetry (ITV), and especially AC Voltammetry (ACV).^52^ ACV is a voltammetric technique operating in the frequency domain, and as such, it allows the separation of the capacitive and resistive contributions (which can be probed at different frequencies of, e.g., applied potential perturbation). As the adsorption of lipids and further AuNPs change dielectric properties of the interfacial region, the monitoring of interfacial capacitance provides indirect insights about their interaction. Figure 3A–C shows the current–potential dependencies recorded after the addition of corresponding lipid (DOTAP, DPSH, and DOPC, respectively) and further AuNPs. The initial set of data shows that DOTAP does not give any signals within the available potential window (this is from around −0.35 to 0.35 V), suggesting a lack of interfacial cationic lipid ion transfer reaction. In turn, the set of peaks from −0.30 V to −0.10 V and elaborated electrochemical response spanning over the entire potential window were recorded for DPSH and DOPC, which indicates that external polarization can trigger the interfacial transfer of both constituents. Since our focus was to study LLI modification with lipids and AuNPs, we moved to ACV, which was found to be a very powerful technique when it comes to probing interfacial adsorption^53–55^ or even direct interfacial charge transfer processes.^56^ ACVs recorded at the low frequency regime (6 Hz) were plotted in the form of the differential capacitance as a function of the Galvani potential difference.^57^ It is important to note that ITIES is polarized in a purely ionic manner (via segregation of the background electrolyte ions that do not partition to the contacting phases within the potential range for which LLI is polarized), and the change in the applied Galvani potential difference value affects the ionic composition on both sides of the interface. This in turn allows us to study potential difference dependent adsorption processes (e.g., for negative polarization direction the aqueous phase side of the ITIES is charged negatively and promotes the adsorption or ion transfer of negatively charged molecules). Moreover, the obtained dependences inform us about the potential of zero charge (the potential at which the net free charge of the interface is the zero—parameter affected by the adsorption of charged constituents) and the amount of charge located at the interface. These two parameters can be used as direct information about interfacial adsorption phenomena and compactness of the formed interfacial deposits. In all studied configurations defined by pure lipids (DOPC, DOTAP or DPSH), lipid mixtures (DOPC-DPSH or DOTAP-DPSH), and finally lipid-AuNPs conjugates, we were expecting to observe and assess the degree of interfacial adsorption governed by the high affinity of the lipids to the biphasic system^58–60^ and further interaction between lipid polar head groups and AuNPs. Figure 3 shows a series of ACVs recorded before and after eLLI modification with lipid monolayers composed of pure lipids and successive AuNPs addition. On top of that, we have also studied two configurations that aimed at the phospholipid monolayer formation. The first set of experiments (Figures 3D,G,J and 4A,D) was done at the pristine eLLI without any additives in both phases, followed by the addition of phospholipids directly to the organic phase, and further AuNPs to the aqueous phase. The second approach (Figures 3E,H,K, and 4B,E) relied on the initial formation of the monolayer at the organic phase surface via the drop casting of a phospholipid-concentrated solution, then covering the formed layer with the aqueous phase, and finally adding AuNPs. Both investigated monolayer formation scenarios led to similar conclusions, while the three investigated phospholipids behaved differently in the biphasic systems, as confirmed by significant changes in the shape of ACVs. The interfacial adsorption of DOTAP to the eLLI could be observed as the change in the *C_dl_* for the $\Delta_{org}^{aq}\Phi$ < 0 V, along with the change in the PZC shifting by 30 mV toward more positive $\Delta_{org}^{aq}\Phi$ . The *C_dl_* increased from 12.3 μF·cm^–2^ for bare eLLI to 15.7 μF·cm^–2^ when DOTAP was added, and further to 18.2 μF·cm^–2^ once AuNPs were present in the aqueous phase (measured at $\Delta_{org}^{aq}\Phi$ = −0.05 V). This indicates that excess charge in the form of DOTAP lipid (with only terminal quaternary ammonium cation existing as the charged polar headgroup) must be related to the interfacial accumulation of chloride anions from the aqueous phase, being in line with the negative polarization of the aqueous phase side of the eLLI (accumulation of anions at the aqueous phase side when the negative $\Delta_{org}^{aq}\Phi$ is applied). Interestingly, the addition of negatively charged AuNPs to the aqueous phase further increased the value of *C_dl_*, indicating their possible adsorption to the polar headgroup of DOTAP driven by electrostatic attraction and the application of negative $\Delta_{org}^{aq}\Phi$ . Interfacial adsorption can also be further confirmed by the lack of ion transfer currents deduced from Figure 3A. When DPSH (see Figure 3G or H) was placed at the eLLI after soft junction formation, the *C_dl_* increased from 16.0 μF·cm^–2^ for bare eLLI to 34.1 μF·cm^–2^ when DPSH was added, and further dropped to 24.0 μF·cm^–2^ in the presence of AuNPs (measured at −0.15 V). Since the appearance of the recorded peaks overlaps with the signals obtained with ITV (see Figure 3B) we have a mixture of two phenomena: (i) interfacial adsorption of DPSH and (ii) its interfacial ion transfer. The drop in differential capacitance at around −0.2 V after AuNPs addition can be explained as the formation of the DPSH-AuNPs conjugate, which, due to its voluminous nature, should not undergo the interfacial ion transfer process. Interestingly, we did not observe sound interaction between DOPC and AuNPs, as shown in Figure 3J and K. The presence of a region with flat *C_dl_* (from −0.2 V to 0 V) indicates stable DOPC monolayer formation,^61^ yet only small changes in the *C_dl_* equal to around 1.7 μF·cm^–2^ found in Figure 3K at −0.1 V after AuNPs addition, indicate that negative charge buildup is very small. In turn, the sharp rise in *C_dl_* > 0.05 V (see Figure 3J) after DOPC addition is attributed to its interfacial ion transfer, also observed on ITVs shown in Figure 3C.

**Figure 3:**
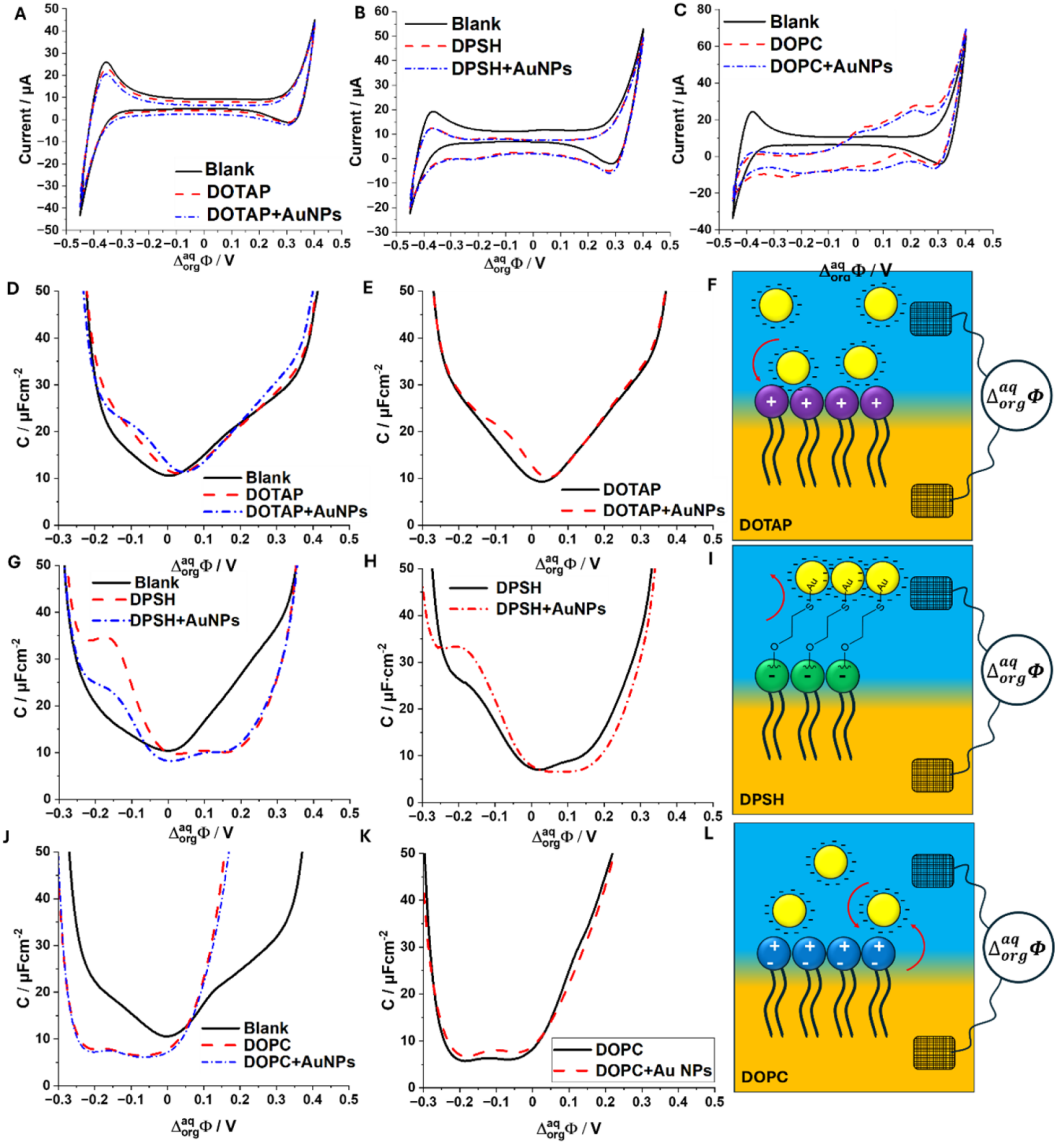
Ion transfer voltammograms (ITVs) recorded in the presence of DOTAP (A), DPSH (B), and DOPC (C) before and after AuNPs addition. AC voltammograms in the left column were recorded before (black solid line) and after the direct addition of the lipid (red dashed line), and further after AuNPs (blue dashed-dotted line) addition to the organic and aqueous phases, respectively (DOTAP – D; DPSH – G; DOPC – J). AC voltammograms in the middle column were recorded first after lipid drop casting over the organic phase, followed by the aqueous phase addition (black solid line), and further after AuNPs (red dashed line) addition to the aqueous phase (DOTAP – E; DPSH – H; DOPC – K). The observed interactions between the lipid monolayer and AuNPs are schematically presented in F – DOTAP, I – DPSH, and L – DOPC.

**Figure 4:**
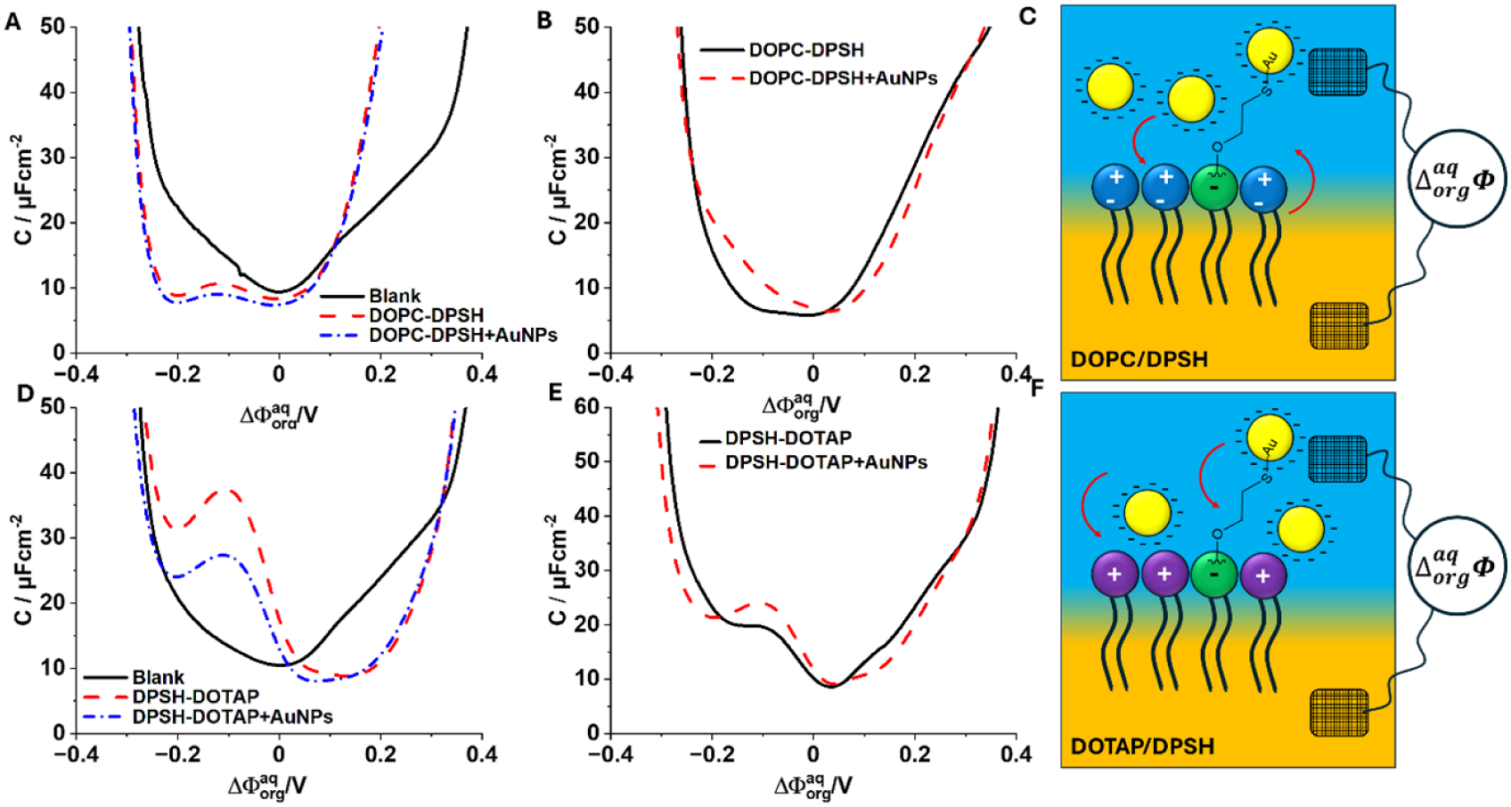
AC voltammograms in the left column were recorded before (black solid line) and after the direct addition of the lipid (red dashed line), and further after AuNPs (blue dash-dotted line) addition to the organic and aqueous phases, respectively (DOPC:DPSH—A; DOTAP:DPSH—D). AC voltammograms in the middle column were recorded first after lipid drop casting over the organic phase, followed by the aqueous phase addition (black solid line), and further after AuNPs (red dashed line) addition to the aqueous phase (DOPC:DPSH – B; DOTAP:DPSH – E). The observed interactions between the lipid monolayer and AuNPs are schematically presented in C – DOPC:DPSH, and F – DOTAP:DPSH.

Once the effect of the monolayer made out of individual types of lipids was examined, we moved and studied the mixtures of lipids, as shown in Figure 4. The presence of DPSH next to DOPC (see Figure 4A and B) resulted in the shape of the ACV resembling what was observed when the eLLI was modified with DOPC only. This means that the latter lipid dominates the interfacial behavior yet still, the addition of AuNPs is visible as a slight increase in the *C_dl_* (from 6.5 to 11.2 μF·cm^–2^) when lipid adsorption occurs prior to eLLI formation (see Figure 4B). This could indicate that the composition of the lipids in the monolayer depends on the interfacial modification method. The composition of the lipid monolayer formed before LLI formation is expected to reflect the initial lipid solution composition. In contrast, the monolayer formed after soft junction formation is additionally influenced by molecular partitioning and the individual affinities of the lipids for the interfacial region. When studying the DOTAP/DPSH mixture, the shape of the ACV curves resembled the dependencies observed for DPSH alone (see Figure 4C and D). This suggests that DPSH plays a significant role in AuNPs binding, while DOTAP, being a lipid that provides an overall positive charge, may further facilitate the adsorption of negatively charged particles, as shown in Figure 4F. The given discussion shows how the analysis of the interfacial capacitance (affected by the charge located within the polar head groups of lipids and the surface charge of AuNPs) and the location of the potential of zero charge may be used to deduce interactions between several constituents adsorbing to the liquid–liquid interface. All our LipoAuNPs formation confirmation results, here supported by the electrochemical investigations, add up and point toward the successful placement of AuNPs at the surface of liposomes that are built from DOTAP, DPSH, and DOPC (the latter used only as the charge-balancing and filling species), which were further used in cell uptake studies.

### 3.3 Liposome Uptake

To thoroughly investigate how modifying the liposome surface with AuNPs affects cellular uptake, two liposome types were compared: DOTAP&DPSH liposomes and DOTAP&DPSH liposomes with AuNPs (LipoAuNPs). Additionally, DPSH liposomes (without AuNPs) were included in the study to assess the influence of lipid composition on cellular interactions. DPSH liposomes modified with AuNPs were not included in the biological studies due to the complexity of the preparation protocol. The cellular internalization of the tested liposomes was evaluated by assessing changes in the fluorescence intensity of cells after various incubation times with carboxyfluorescein-labeled liposomes. The results obtained are presented in Figure 5.

**Figure 5:**
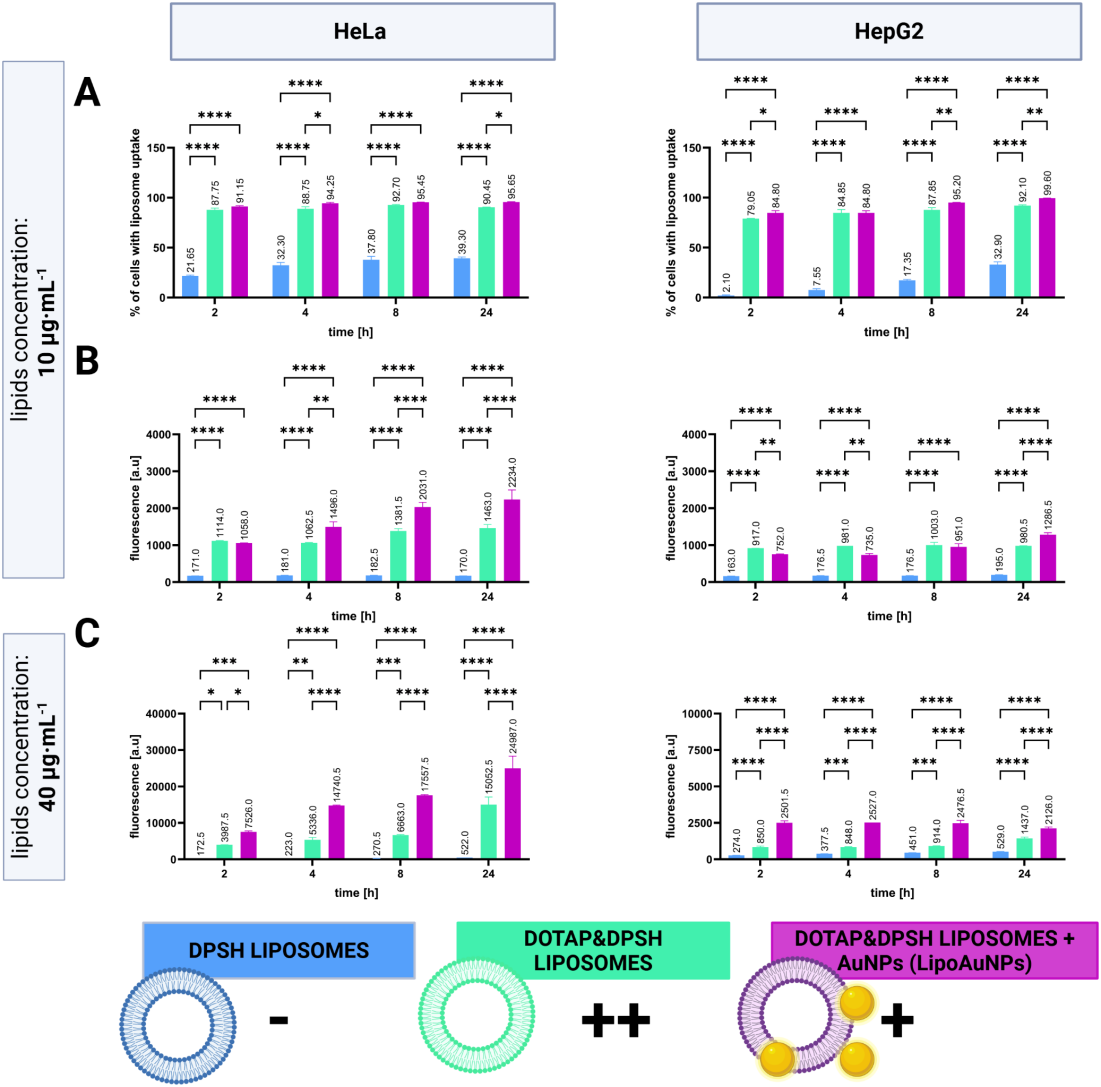
Cellular uptake of DPSH liposomes, DOTAP&DPSH liposomes, and DOTAP&DPSH liposomes with AuNPs for the HeLa cell line and HepG2 cell line after incubation for 2, 4, 8, and 24 h. All results are presented as the average of two independent measurements ± standard deviation. Each measurement is an average of 10000 cells. Asterisks indicate the level of statistical significance for differences between the results obtained with different types of liposomes at various time points (ns: *p* > 0.05; *: *p* ≤ 0.05; **: *p* ≤ 0.01; ***: *p* ≤ 0.001; ****: *p* ≤ 0.0001). (A) Results are presented as % of cells with liposome uptake for a lipid concentration of 10 μg·mL^‑1^. (B) Results are presented as mean fluorescence for a lipid concentration of 10 μg·mL^‑1^. (C) Results are presented as mean fluorescence for a lipid concentration of 40 μg·mL^‑1^.

The change in the percentage of cells with liposome uptake over time is presented in Figure 5A. It represents the % of cells that exhibit higher fluorescence values than the control sample (i.e., higher than the autofluorescence of cells that were not incubated with fluorescent liposomes). Note that carboxyfluorescein was covalently attached to the lipids within the bilayer, meaning that the observed signal reflects carrier uptake and not content release. In the case of DPSH liposomes, it can be observed that their internalization occurs very slowly compared to other variants. At the lipid concentration of 10 μg·mL^–1^ in cell culture medium, in the case of the HeLa cell line, only 21.65% of cells were characterized by liposome uptake after 2 h. After 24 h, the uptake level was still relatively low, i.e., 39.30%. In the case of HepG2 cells, analogous findings were even lower: 2.10% after 2 h and 32.90% after 24 h. Bare DOTAP&DPSH liposomes and LipoAuNPs demonstrated very high and rapid internalization in both HeLa and HepG2 cells. After 2 h of incubation with DOTAP&DPSH liposomes over 87% of HeLa cells and 79% of HepG2 cells exhibited a fluorescence signal. For LipoAuNPs, these values were even higher—91.15% for HeLa and 84.8% for HepG2. At subsequent time points, the percentage of cells with internalized DOTAP&DPSH liposomes and LipoAuNPs remained high or increased slightly, reaching values above 90% and reaching 99.6% for HepG2 cells internalizing LipoAuNPs after 24 h.

To comprehensively assess liposome internalization, we analyzed both the percentage of cells exhibiting a fluorescence signal above background levels (% of cells with liposome uptake) and the mean fluorescence intensity per cell (mean fluorescence). The former allows us to assess the extent of the effect in the cell population, while the latter reflects the amount of liposomes absorbed by individual cells. Combining both approaches allows for a more precise interpretation of the effectiveness of intracellular liposome delivery. Figure 5B and C present the mean fluorescence levels exhibited by cells after incubation from 2 to 24 h with different liposome types at two lipid concentrations: 10 and 40 μg·mL^–1^.

For DPSH liposomes, the mean fluorescence level results are significantly lower than those for the other variants. Among the three tested liposome variants, LipoAuNPs generated the highest mean fluorescence values, especially for the HeLa cell line after 24 h. Note that the differences in the mean fluorescence levels between cells incubated with DOTAP&DPSH liposomes and LipoAuNPs developed over time and were greater at higher lipid concentrations in the cell culture medium. At the initial time points (2–4 h), the differences in the mean fluorescence of cells incubated with DOTAP&DPSH liposomes and LipoAuNPs were moderate, or values were even slightly higher for bare liposomes. For instance, for HeLa at a concentration of 10 μg·mL^–1^ and 2 h of incubation, these values were ∼1100 au for DOTAP&DPSH liposomes and ∼1000 au for LipoAuNPs. However, with increasing incubation time, cells incubated with LipoAuNPs exhibited a significantly greater increase in fluorescence signal than those with DOTAP&DPSH liposomes. After 24 h, for DOTAP&DPSH liposomes the mean fluorescence values are ∼1500 au at 10 μg·mL^–1^ and ∼15000 au at 40 μg·mL^–1^. For LipoAuNPs, these values are much higher, i.e., ∼2200 au and ∼25000 au, respectively. Interestingly, the difference in the average fluorescence level between cells incubated with LipoAuNPs and bare liposomes appears much earlier at higher tested lipid concentrations (40 μg·mL^–1^). In the case of the HeLa cell line, after just 2 h, the difference between DOTAP&DPSH liposomes (∼4000 au) and LipoAuNPs (∼7500 au) is very clear. For the HepG2 cell line and a lipid concentration of 10 μg·mL^–1^, the differences between DOTAP&DPSH liposomes and LipoAuNPs are smaller, but LipoAuNPs still reach higher levels (e.g., 980.5 au and 1285.5 au). An analogous tendency regarding the effect of concentration on differences between liposome variants can be observed for the HepG2 cell line. After 24 h, at a concentration of 40 μg·mL^–1^ the differences between DOTAP&DPSH liposomes (∼1437 au) and LipoAuNPs (∼2126 au) are evident.

To confirm that the higher mean fluorescence results for LipoAuNPs are not the result of AuNPs autofluorescence, the fluorescence of both liposome variants with lipids with carboxyfluorescein was measured. At an excitation wavelength of 490 nm, the emission peak occurred at 530 nm (see Figure S10 in Supporting Information). AuNPs not only fail to enhance the fluorescence of carboxyfluorescein but also cause a slight quenching effect. Therefore, AuNPs autofluorescence can be excluded, and is in line with what others have reported.^62^ The fact that the cells showing higher fluorescence were those incubated with liposomes with a slightly lower fluorescence level indicates that the difference in fluorescence levels in favor of the LipoAuNPs variant is credible.

To analyze whether the observed fluorescence in both cases originates from fully internalized liposomes, flow cytometry with trypan blue addition was performed. First, carboxyfluorescein quenching was demonstrated experimentally (Figure S3). This phenomenon is also well documented in the scientific literature.^39,63^ Trypan blue was added to the live cells immediately before flow cytometry measurements, as described in the materials section. Since trypan blue is actively removed from live cells, it only quenches external fluorescence. Quenching of external cell fluorescence was performed for the HepG2 cell line at a lipid concentration of 40 μg·mL^–1^. As presented in Figure S11, adding trypan blue did not reverse the previously observed trends—cells incubated with LipoAuNPs remained the variant showing the highest fluorescence. Notably, after the extrinsic fluorescence was quenched, the average values decreased for all variants.

In the case of DPSH liposomes, both the percentage of cells with liposome uptake and the mean fluorescence level are completely consistent with each other and confirm that DPSH liposomes are the least rapidly internalized among the tested variants. This tendency is not surprising and is most likely due to the fact that DPSH liposomes, contrary to other tested nanoobjects, have a negatively charged surface.^51,64,65^ LipoAuNPs are characterized by a slightly higher percentage of cells with liposome uptake than bare DOTAP&DPSH liposomes. This result is intriguing since bare DOTAP&DPSH liposomes hold a much higher surface charge value and consequently higher affinity to cell membranes than the variant with AuNPs (38.1 ± 2.2 mV and 14.67 ± 0.2 mV at pH 7.0, respectively). However, as described in an elegant work by Forest and Pourchez, taking into account only the surface charge of nanoparticles is an overly simplistic explanation; other factors such as nanoparticle size, shape, surface chemical functions, and protein corona should be taken into consideration.^66^ Mechanistically, the higher uptake of LipoAuNPs despite their lower average ζ-potential suggests that cellular association is not governed solely by the mean surface charge. AuNP decoration is expected to introduce nanoscale chemical and electrostatic heterogeneity. Such local charge-density/chemistry patterns can increase avidity to the negatively charged glycocalyx and membrane components and thereby promote adsorptive interactions, even when the ensemble-averaged ζ-potential is reduced. In addition, the presence of surface-exposed Au is likely to modulate the adsorption of serum proteins and the composition of the protein corona. In practice, corona formation may (i) mask or reshape electrostatic interactions at the interface, (ii) provide ligands enabling receptor-assisted internalization, and (iii) bias the endocytic route toward pathways with a distinct intracellular fate. These effects provide a plausible explanation for the consistently higher mean cellular fluorescence observed for LipoAuNPs relative to that of the uncoated liposomes, particularly at longer incubation times.

When comparing DOTAP&DPSH liposomes with their AuNP counterparts, the mean fluorescence level measurements reveal a trend that was hidden in the data presented as a percentage of cells with liposome uptake. In this case, the differences between DOTAP&DPSH liposomes and LipoAuNPs are significantly higher. Additionally, when analyzing the mean fluorescence level results, two very interesting phenomena should be noted. The difference was much more pronounced for higher concentrations of the nanosystems in the cellular medium. The quenching of extrinsic fluorescence (originating from liposomes that only coat the cells from the outside) presented in Figure S11 did not reverse the trend, and LipoAuNPs remained the variant most intensively internalized by cells. However, this experiment showed that, in the case of LipoAuNPs, the high fluorescence value observed for short incubation times (for samples with lipid concentrations of 40 μg·mL^–1^) originates from liposomes that strongly coat the cells from the outside and only gradually internalize over time.

Furthermore, the differences in the mean fluorescence levels of cells incubated with DOTAP&DPSH liposomes and LipoAuNPs deepened with increasing incubation time in the HeLa cell line. This may mean that, in addition to higher internalization, LipoAuNPs accumulate more effectively over time or follow a different intracellular pathway. The hypothesis of different cellular trafficking of these variants makes particular sense, considering the properties of the fluorescent marker used. Specifically, carboxyfluorescein is a compound that loses its fluorescent properties in an acidic environment due to the protonation of carboxylic acid and phenol groups.^67^ Inside a cell, the most acidic compartment is the lysosome, where the pH can drop to around 5.^68^ Other acidic regions include endosomes, which have a pH range of 5 to 6, and the lumens of secretory organelles, where the pH gradually decreases along the secretory pathway.^68^ For this reason, the difference in mean fluorescence levels may be particularly large after 24 h because it is caused by a combination of two phenomena: different uptake intensities and different cellular trafficking (e.g., one variant being more readily absorbed into the acidic cellular environment, where carboxyfluorescein fluorescence is partially suppressed). To test the hypothesis that LipoAuNPs exhibit different cellular trafficking, a detailed confocal microscopy analysis was performed.

While studies addressing the influence of gold nanostructures on liposome cellular uptake have been reported, direct comparisons performed on analogous AuNP–liposome architectures are still limited. In particular, systematic investigations of preformed AuNPs covalently tethered to liposomes via thiol-containing lipids have not been extensively explored. Nevertheless, previous studies have investigated alternative gold–lipid architectures and their role in promoting cellular uptake, which are discussed below for comparison.

For example, Shahabi et al. investigated the cytotoxicity of a free drug (hydroxyurea), its liposomal formulation, liposomes combined with AuNPs, and liposomal AuNPs additionally complexed with DNA in MCF-7 cells. All liposomal formulations exhibited higher cytotoxicity than the free drug. While no significant differences were observed between liposomal variants at higher drug concentrations, at the lowest tested concentrations (5–10 μM), the formulation combining liposomes, AuNPs, and DNA showed the strongest cytotoxic effect. These results indirectly suggest that the presence of AuNPs in liposomal systems may facilitate intracellular delivery of nucleic acids; however, cellular uptake was not directly quantified in that study.^69^ One of the most closely related studies was reported by Wang et al. in 2022, who developed a fluidity-guided liposomal nanomotor system based on gold core–platinum shell nanoparticles (Au@Pt) assembled on the surface of liposomes.^70^ In this work, the assembly and spatial distribution of Au@Pt on liposomal membranes were controlled by tuning the lipid composition and membrane fluidity. Importantly, Au@Pt retained catalase-like activity upon surface attachment, enabling the decomposition of H_2_O_2_ and resulting in autonomous motion of the liposomal nanomotors. As a consequence, enhanced cellular uptake was observed in HepG2 cancer cells; however, this effect was strictly dependent on the presence of H_2_O_2_ in the culture medium. Therefore, the increased uptake reported in this study should be attributed to the active, catalase-driven propulsion of the liposomal nanomotors rather than the passive influence of metal nanoparticle decoration alone.

Previous studies have demonstrated that subtle modifications of the nanoparticle surface can dramatically influence cellular uptake. In particular, Ho et al.^71^ reported that partial alkylation of PEGylated AuNPs significantly enhanced their internalization by Kera-308 keratinocytes. The authors showed that introducing a small fraction of dodecyl- or octadecyl-terminated PEG (0.2 wt %) increased cellular uptake up to 30-fold compared to methoxy-PEG-AuNPs or hexyl-terminated analogues after 24 h of incubation. These findings highlighted that even minimal variations in surface chemistry, such as alkyl chain length and density, can strongly modulate nanoparticle–cell interactions. In a subsequent study, the same group demonstrated that dodecyl-terminated PEG-coated AuNPs not only exhibited enhanced uptake but also altered intracellular trafficking and exocytosis pathways in a composition-dependent manner, further emphasizing the sensitivity of biological responses to surface functionalization.^72^ In contrast to these approaches, where lipid-like moieties are conjugated directly to the AuNP surface to promote uptake, our strategy is based on attaching AuNPs to preformed liposomes. This design preserves the structural and functional versatility of liposomal carriers while introducing the unique physicochemical properties of gold nanoparticles. Although in our uptake studies the quantified signal corresponds to fluorescently labeled lipids rather than gold itself, the enhanced internalization observed for LipoAuNPs indicates that surface decoration of liposomes with AuNPs represents an alternative and complementary strategy to nanoparticle alkylation for modulating cellular entry. Together, these studies underscore that both nanoparticle-centered and liposome-centered design strategies can effectively regulate nano–bio interactions.

### 3.4 Liposome Cellular Trafficking

To further investigate the influence of AuNPs on the internalization process and cellular trafficking, we employed confocal microscopy. Cells were stained with DAPI to visualize the nuclei and with CellMask Deep Red to outline the plasma membrane. Representative images are presented in Figure S12 (HeLa cells) and Figure S13 (HepG2 cells) in the Supporting Information. The images revealed that both bare DOTAP&DPSH liposomes and LipoAuNPs accumulate primarily in the peripheral regions of the cytosol. Confocal microscopy further confirmed, in agreement with flow cytometry data, that cells incubated with LipoAuNPs exhibit greater fluorescence compared to their uncoated variant. This effect was more pronounced in HepG2 cells than in HeLa cells. To determine whether bare DOTAP&DPSH liposomes are more likely to be transported into acidic intracellular compartments than LipoAuNPs (as suggested by the lower fluorescence observed after incubation), staining and imaging were performed using LysoTracker (see Figures 6 and 7). In these images, nuclei were visualized with DAPI (column 1), liposomes with carboxyfluorescein-labeled lipids (column 2), and acidic compartments with LysoTracker DeepRed (column 3). Column 4 shows merged images of DAPI, FAM, and LysoTracker Deep Red, whereas column 5 presents 3D reconstructions of the same channels, enabling visualization of spatial relationships between signals.

**Figure 6:**
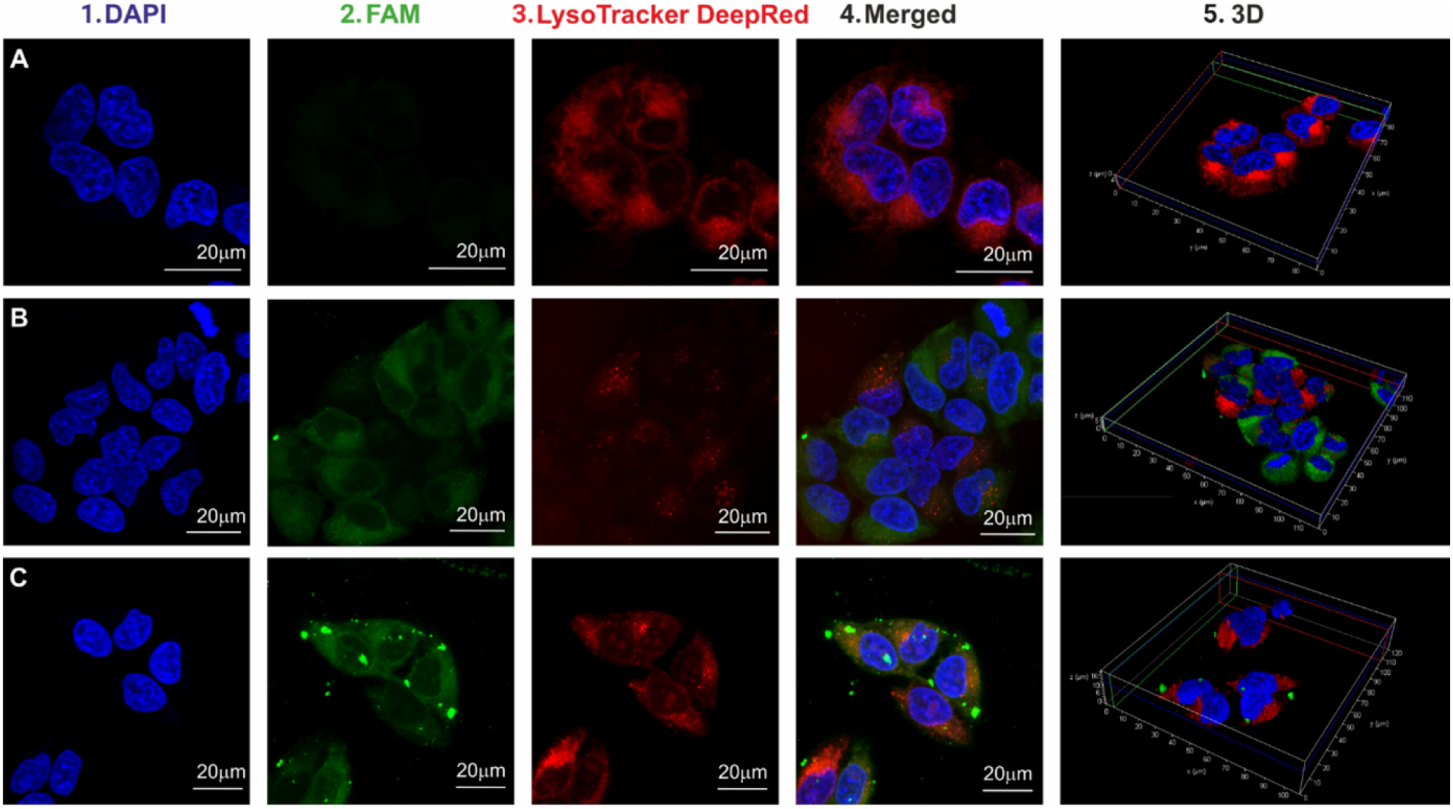
Confocal images of **HeLa** cells: (**row A**) untreated, (**row B**) treated with DOTAP&DPSH liposomes, and (**row C**) treated with LipoAuNPs. Cells were incubated with liposomes at a lipid concentration of 10 μg·mL^–1^ for 4 h. Cell nuclei were stained with DAPI (**column 1**), liposomes were visualized via carboxyfluorescein (FAM)-labeled lipids (**column 2**), and acidic compartments were visualized using LysoTracker Deep Red (**column 3**). **Column 4** shows merged images of DAPI, FAM, and LysoTracker Deep Red, while **column 5** presents 3D reconstructions based on the same channels.

**Figure 7:**
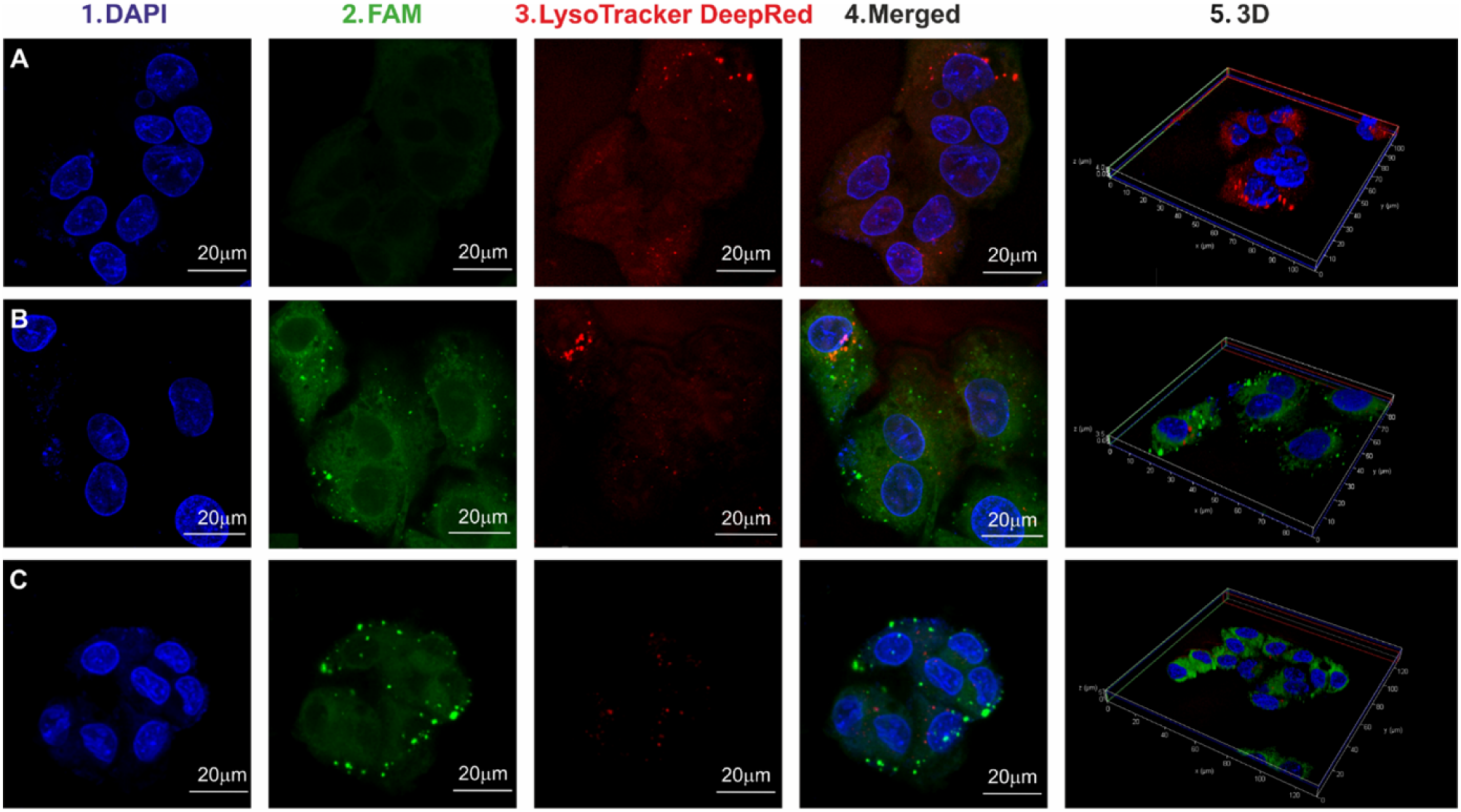
Confocal images of **HepG2** cells: (**row A**) untreated, (**row B**) treated with DOTAP&DPSH liposomes, and (**row C**) treated with LipoAuNPs. Cells were incubated with liposomes at a lipid concentration of 10 μg·mL^–1^ for 4 h. Cell nuclei were stained with DAPI (**column 1**), liposomes were visualized via carboxyfluorescein (FAM)-labeled lipids (**column 2**), and acidic compartments were visualized using LysoTracker Deep Red (**column 3**). **Column 4** shows merged images of DAPI, FAM, and LysoTracker Deep Red, while **column 5** presents 3D reconstructions based on the same channels.

Based on the LysoTracker-stained images (Figures 6 and 7, column 3), the colocalization of fluorescently labeled liposomes (column 2) with lysosomes and late endosomes was quantified. Colocalization analysis (Table 3) revealed a substantial reduction in the interaction between liposomes and lysosomes upon AuNPs coating. In HeLa cells, the colocalization rate decreased approximately 5-fold (from 20.85 ± 7.20% to 4.00 ± 2.35%), while in HepG2 cells, it dropped about 3-fold (from 11.08 ± 2.34% to 4.01 ± 1.90%). This was further supported by declines in both Pearson’s correlation and overlap coefficient values. These findings indicate that functionalization with AuNPs significantly alters the intracellular trafficking of liposomes, reducing their accumulation in lysosomes.

**Table 3:** Correlation of the Colocalization of the Tested Liposomes with Lysosomes for the HeLa and HepG2 Cell Lines^a^

Parameter	Nanoobject type	HeLa	HepG2
Pearson’s Correlation	DOTAP&DPSH Liposomes	0.500 ± 0.243	0.187 ± 0.083
	DOTAP&DPSH Liposomes + AuNPs	0.274 ± 0.085	0.282 ± 0.111
Overlap Coefficient	DOTAP&DPSH Liposomes	0.6695 ± 0.096	0.5053 ± 0.136
	DOTAP&DPSH Liposomes + AuNPs	0.3838 ± 0.086	0.407 ± 0.139
Colocalization Rate	DOTAP&DPSH Liposomes	20.85 ± 7.20%	11.08 ± 2.34%
	DOTAP&DPSH Liposomes + AuNPs	4.00 ± 2.35%	4.01 ± 1.90%

a Calculations Were Made on the Basis of Confocal Microscopy. Cells Were Incubated with Liposomes 10 μg·mL^–1^ for 4h.

Imaging using CellMask Deep Red did not explain the difference in the average fluorescence level between DOTAP&DPSH liposomes and LipoAuNPs obtained by flow cytometry. However, staining with LysoTracker Deep Red, which labels acidic compartments such as late endosomes and lysosomes, provided very interesting insights. We found that DOTAP&DPSH liposomes exhibited higher colocalization with late endosomes and lysosomes than LipoAuNPs. This is consistent with the differences in mean fluorescence levels, which increased over time. Additionally, the differences in colocalization with lysosomes between the HeLa and HepG2 cell lines are perfectly consistent with the differences in the average fluorescence levels in these lines. In the case of the HeLa cell line, the differences in the mean fluorescence level between cells incubated with bare DOTAP&DPSH liposomes were higher than in the case of the HepG2 cell line. Similarly, a greater difference in colocalization with acidic compartments was observed in HeLa cells compared to HepG2 cells. Reduced colocalization with LysoTracker-positive acidic compartments can arise from several nonmutually exclusive scenarios: (i) diversion to nonacidic/recycling pathways, (ii) delayed endosomal maturation and/or reduced acidification, and/or (iii) increased release of the liposomal signal from endosomal compartments (“endosomal escape”). In our system, this interpretation is further informed by the use of carboxyfluorescein-labeled liposomes, because carboxyfluorescein fluorescence is attenuated under acidic conditions. Therefore, the time-dependent divergence in mean cellular fluorescence, together with the markedly lower LysoTracker colocalization of LipoAuNPs, is consistent with reduced delivery to acidic late endosomes/lysosomes and suggests more efficient persistence in less acidic compartments and/or increased escape prior to lysosomal degradation. We acknowledge that direct assays of endosomal membrane damage or cytosolic cargo release would be required to unambiguously confirm membrane rupture; nevertheless, our quantitative confocal analysis demonstrates a clear AuNP-dependent shift in intracellular processing away from acidic compartments.

Such behavior is very beneficial from the point of view of biomedical applicability, as it relates to the ability of therapeutic agents, such as drugs, nucleic acids, or nanoparticle-based delivery systems, to exit endosomal compartments after endocytosis and before their maturation into late endosomes or fusion with lysosomes. This step is crucial for ensuring that the cargo reaches its intracellular target, particularly in the cytosol, and avoids degradation in acidic and enzyme-rich lysosomal environments.^19,73^ Importantly, this feature is lacking in widely used second-generation PEGylated liposomes. While PEG chains effectively reduce premature drug release and prolong systemic circulation, they may simultaneously impair cellular internalization and endosomal escape. This limitation, commonly referred to as the “PEG dilemma,” highlights the need for alternative surface engineering strategies that balance circulation stability with efficient intracellular delivery.^74,75^

### 3.5 Liposome Cytotoxicity

The next important step to confirm the biomedical potential of LipoAuNPs was to check whether modification with AuNPs influences cell viability. For this purpose, the MTT assay was used, as shown in Figure 8A for HeLa and Figure 8B for HepG2 cell lines. Statistical analysis (Table S1) showed that, for all tested liposome types, the lipid concentration does not affect cell viability in the HepG2 cell line. In the case of the HeLa cell line, higher concentrations reduced cell viability, but the decrease was limited to 70% at a high lipid concentration of 200 μg·mL^–1^. To further confirm these results and eliminate the potential interference of AuNPs with a spectroscopic cell viability assay, real-time monitoring of cell viability with xCELLigence technology was used. This method is based on measuring the impedance between Au microelectrodes integrated at the bottom of the culture wells. Briefly, as cells adhere, spread, and proliferate, the impedance increases. Based on these impedance readings, a cell growth curve is generated over time. The obtained growth curves (see Figure S14 in the Supporting Information) are consistent with the MTT test. For the HepG2 line, the growth curve of cells incubated with liposomes did not differ from that of control cells. In contrast, in the HeLa cell line, on all growth curves for cells incubated with all tested liposomes (DPSH Liposomes, DOTAP&DPSH Liposomes, and DOTAP&DPSH Liposomes with AuNPs), a distinct flattening of the growth curve was observed, which was not seen in the control. This indicates that higher concentrations of the tested liposomes cause a transient inhibition of the proliferation of HeLa cells.

**Figure 8:**
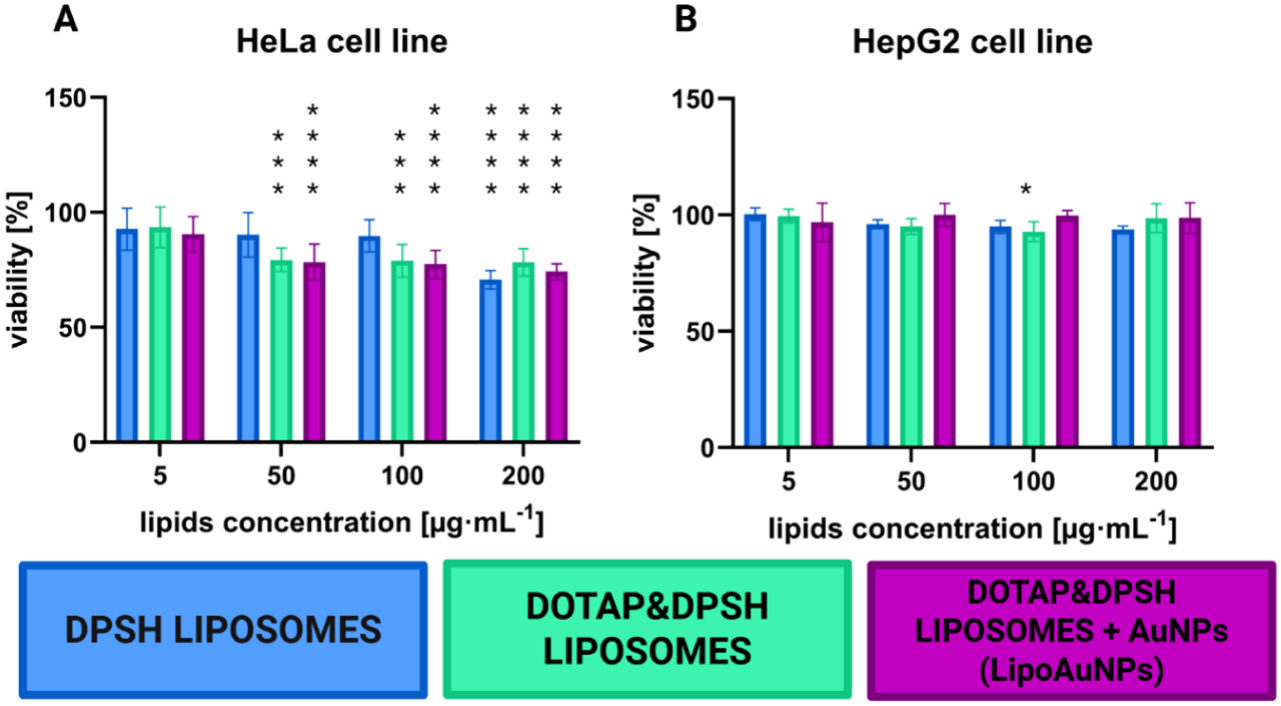
Viability of HeLa (**A**) and HepG2 (**B**) cells after 24 h of incubation with liposomes at various lipid concentrations (5, 50, 100, and 200 μg·mL^–1^) assessed using the MTT assay. Cell viability was normalized to untreated control cells (100%). Data are presented as mean ± SD from six independent experiments, with statistical significance compared to the untreated control (ns: *p* > 0.05; *: *p* ≤ 0.05; **: *p* ≤ 0.01; ***: *p* ≤ 0.001; ****: *p* ≤ 0.0001).

A key observation is that neither the surface charge (comparison between DPSH liposomes and DOTAP&DPSH liposomes) nor the attachment of AuNPs (comparison between liposomes with and without AuNPs) affects cell viability. All tested liposome variants showed no cytotoxicity within the examined concentration range in the HepG2 cell line. In the HeLa cell line, a slight decrease in cell viability was observed with increasing concentrations. The higher cytotoxicity values obtained for the HeLa line are consistent with the cellular uptake measurement results, which showed that HeLa internalizes liposomes more intensively than the HepG2 line. Moreover, the obtained results regarding cell viability are consistent with other literature reports, in which the lack of effect of AuNPs on the cytotoxicity of liposomes was confirmed.^76–78^

The developed LipoAuNPs platform demonstrates several features supporting its potential applicability in translational nanomedicine. First, the rational lipid composition (DOTAP/DPSH/DOPC) enables controlled and reproducible one-pot attachment of citrate-stabilized AuNPs without additional nanoparticle or liposome prefunctionalization, which simplifies manufacturing and facilitates scalability. The combined electrostatic preorganization and covalent Au–S anchoring ensure structural stability under biologically relevant conditions, reducing the risk of nanoparticle detachment in complex media. From a therapeutic perspective, integrating AuNPs with a biodegradable liposomal scaffold may help mitigate the limitations associated with free AuNPs. Moreover, surface-bound AuNPs retain their plasmonic properties, opening possibilities for multimodal applications such as photothermal therapy or imaging, while the liposomal core preserves the drug encapsulation capability. Liposomes modified with AuNPs via *ex situ* approaches have been previously investigated for applications in drug delivery, imaging, and biosensing.^28,79–81^ A key advantage of our optimized system, particularly in the context of drug delivery, is its ability to promote efficient cellular internalization without detectable cytotoxicity within the tested concentration range. Importantly, AuNP decoration resulted in enhanced cellular uptake accompanied by reduced colocalization with LysoTracker-positive compartments, suggesting altered intracellular trafficking. Altogether, the proposed system represents a structurally defined, scalable, and functionally versatile platform that may serve as a foundation for future drug-loaded therapies, PTT, and thermal imaging.

## 4 Conclusions

In this work, we performed a comprehensive study aimed at understanding and defining the best formulation of lipids promoting the adsorption of citrate-stabilized AuNPs to obtain LipoAuNPs for use in cancer therapy. First, we found that the electrostatic interactions between negatively charged AuNPs and the surface charge of the liposomes (governed by the polar head groups) play a significant role in metallic particle interfacial absorption, even in the presence of thiol-terminated phospholipid. Then, we developed a new lipid mass percentage composition allowing for effective, one-step, and one-pot attachment of AuNPs to the liposome surface. This solution is based on a combination of two approaches: electrostatic attraction of negatively charged AuNPs by the positively charged liposomes with incorporated cationic lipids and binding via covalent bonds due to the presence of thiol-containing lipids. The optimal lipid proportion we developed is as follows: 25% DOTAP (providing the entire nanosystem with a positive surface charge), 10% DPSH (providing the nanosystem with thiol groups that covalently bind to AuNPs), and 63% DOPC (charge-balancing lipid). Additionally, we used 2% PC CF—a lipid with carboxyfluorescein to track the system in cells.

Next, we proposed a novel, multistep experimental approach aiming to confirm the presence of AuNPs on the liposome surface. In this respect, we used dynamic light scattering, zeta potential measurement, electrified liquid–liquid interface ion transfer, alternative current voltammetry measurements, and finally, transmission electron microscopy imaging. The first quality control factor is an increase in the mean hydrodynamic diameter of liposomes after mixing them with AuNPs, corresponding to the diameter of approximately two AuNPs. The second method developed to quickly confirm the adsorption of AuNPs to the liposome surface is to spectrophotometrically confirm the shift of the absorbance peak of gold nanoparticles upon attachment to liposomes. In our case, after the adsorption, we observed a 10 nm shift in the *λ*_*max*_.

Lastly, we thoroughly examined the biological activity of the obtained LipoAuNPs on two cell lines, confirming their high applicability in medicine. Cellular uptake studies using flow cytometry have shown that attaching AuNPs to the surface of liposomes increases their internalization by cancer cells. This result was intriguing since bare DOTAP&DPSH liposomes have a much higher surface charge and consequently higher affinity to cell membranes than the variant with AuNPs. The difference in mean fluorescence for DOTAP&DPSH liposomes and DOTAP&DPSH liposomes with AuNPs becomes particularly apparent after 24 h of incubation, which led to the hypothesis that LipoAuNPs undergo different cellular trafficking than bare DOTAP&DPSH liposomes. Therefore, we decided to compare this by thoroughly analyzing cellular trafficking by confocal microscopy. Colocalization analysis of liposomes with acidic cellular compartments (e.g., lysosomes and late endosomes) by confocal microscopy revealed a substantial reduction in the colocalization of liposomes with acidic compartments upon AuNP coating. Such behavior is very beneficial from the point of view of biomedical applicability because it shows that covering liposomes with gold nanoparticles may help them to avoid endosomal entrapment. All tested liposome variants showed no cytotoxicity within the examined concentration range in the HepG2 cell line. In the HeLa cell line, a slight decrease in cell viability was observed with increasing concentrations. However, this effect occurred with both bare liposomes and liposome–gold nanoparticle conjugates, indicating that the presence of gold does not contribute to increased toxicity of the nanosystems.

## Supplementary Material



## Notes

In the Supporting Information, provided as a separate file, we include figures and tables that complement the results presented in the main manuscript and provide additional evidence for the described findings. The following is a list of all elements included in the Supporting Information: Schematic illustration of the experimental setup for electrode polarization and AC voltammetry measurements (**Figure S1**); flow cytometry gating strategy for evaluation of cellular fluorescence after liposome incubation (**Figure S2**); decrease in fluorescence of carboxyfluorescein-labeled liposomes upon trypan blue addition (**Figure S3**); dynamic light scattering-based size distribution of LipoAuNPs for DPSH (**Figure S4**) and DOTAP&DPSH liposomes (**Figure S5**); TEM micrographs and size distribution of citrate-stabilized AuNPs (**Figure S6**); control experiment showing AuNP aggregation in an environment without liposomes (**Figure S7**); SEM-EDX analysis of liposome binding on Au-coated Cu wires (**Figure S8**); assessment of LipoAuNPs stability over time based on hydrodynamic diameter and PDI measurements (**Figure S9**); fluorescence emission of carboxyfluorescein-labeled liposomes in the presence and absence of AuNPs, showing quenching effects due to AuNP incorporation (**Figure S10**); cellular uptake measurements with trypan blue quenching to exclude fluorescence from liposomes adsorbed on the cell surface (**Figure S11**); confocal images showing liposome uptake in HeLa (**Figure S12**) and HepG2 cells (**Figure S13**); statistical analysis of liposome cytotoxicity (**Table S1**); and real-time cell viability monitoring using impedance measurements (**Figure S14**) (PDF)
