# Supplementary material for: Novel Gold-Functionalization of Liposomes and Its Impact on Cellular Uptake and Trafficking
Source: Mol Pharm. 2026 Apr 10;23(5):2898–917. doi: 10.1021/acs.molpharmaceut.5c01722 (PMC13298881; doi:10.1021/acs.molpharmaceut.5c01722)
Supplement: Supplementary file 1 [file mp5c01722_si_001.pdf]

Supporting Information

Novel gold-functionalization of liposomes and its impact on cellular uptake and trafficking

Agata Margielewska,[a,b] Klaudia Łuków,[a] Abdelatif Laroui,[c,d] Monika Marcinkowska,[a] Sylwia Michlewska,[e] Michał Gorzkiewicz,[a,f] Łukasz Półtorak,[c,*] Barbara Klajnert-Maculewicz[a,**]

[a] University of Lodz, Faculty of Biology and Environmental Protection, Department of General Biophysics, 141/143 Pomorska St., 90-236 Lodz,Poland

[b] Bio-Med-Chem Doctoral School of the University of Lodz and Lodz Institutes of the Polish Academy of Sciences, University of Lodz, 12/16 Banacha St., 90-237 Lodz, Poland

[c] University of Lodz, Electrochemistry@Soft Interfaces (E@SI) Team, Department of Inorganic and Analytical Chemistry, Faculty of Chemistry, 12 Tamka St. 91-403, Lodz, Poland

[d] Doctoral School of Exact and Natural Sciences, University of Lodz, 12/16 Banacha St., 90-237 Lodz, Poland

[e] University of Lodz, Faculty of Biology and Environmental Protection, Laboratory of Microscopic Imaging and Specialized Biological Techniques, 12/16 Banacha St., 90-237 Lodz, Poland

[f] Department of Molecular Medicine II, Medical Faculty and University Hospital, Heinrich Heine University Düsseldorf, Universitätsstr. 1, 40225 Düsseldorf, Germany

[*] lukasz.poltorak@chemia.uni.lodz.pl

[**] barbara.klajnert@biol.uni.lodz.pl

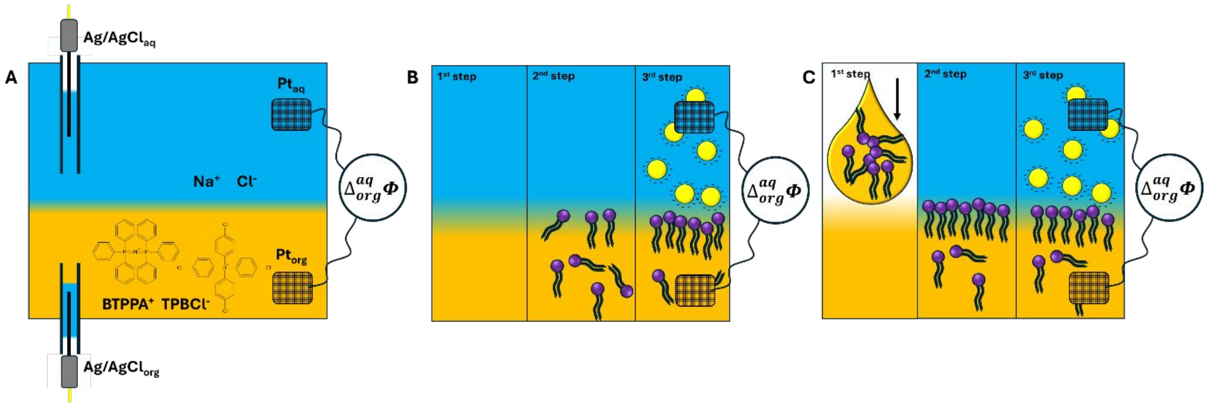

Figure S1. *- A – the schematic representation of the four electrode configuration used to polarize the water (Tris, sodium chloride solution) // 1,2-dichloroethane (BTPPATPBCl solution). Reference and counter electrodes were made out of Ag/AgCl and Pt, respectively. The AC voltammograms were recorded in a particular sequence of lipids and AuNPs addition. Part B-B shows experiments where the AC voltammograms were recorded in the absence of AuNPs and lipids (1st step), after the addition of lipids (2nd step) and finally after the addition of AuNPs. Part BC shows experiments where first the organic phase surface was modified with a lipid layer by drop casting the lipids dissolved in 1,2-DCE over the organic phase (1st step) followed by the addition of the aqueous phase (2nd step), and finally AuNPs (3rd step).*

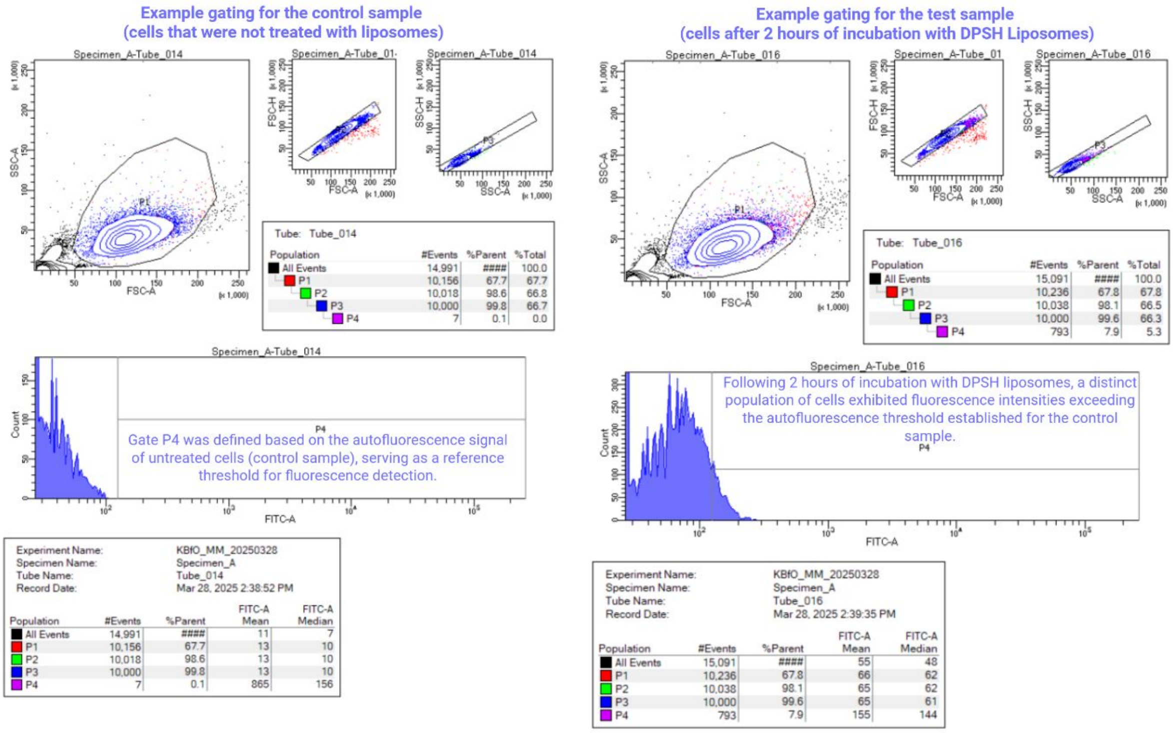

Figure S2. *Representative flow cytometry gating strategy for evaluation of cellular fluorescence after incubation with DPSH liposomes. Left panels: Control sample (untreated cells). Gate P4 was defined based on the baseline autofluorescence signal of cells, used to distinguish fluorescence-positive events (% of cells with liposome uptake). Right panels: Test sample after 2 hours of incubation with DPSH liposomes. A subpopulation of cells exceeded the autofluorescence threshold (Gate P4). Histograms represent FITC-A fluorescence intensity distribution. Dot plots illustrate forward and side scatter gating (FSC-A/SSC-A). Quantitative data on population distribution are shown in tables below each panel.*

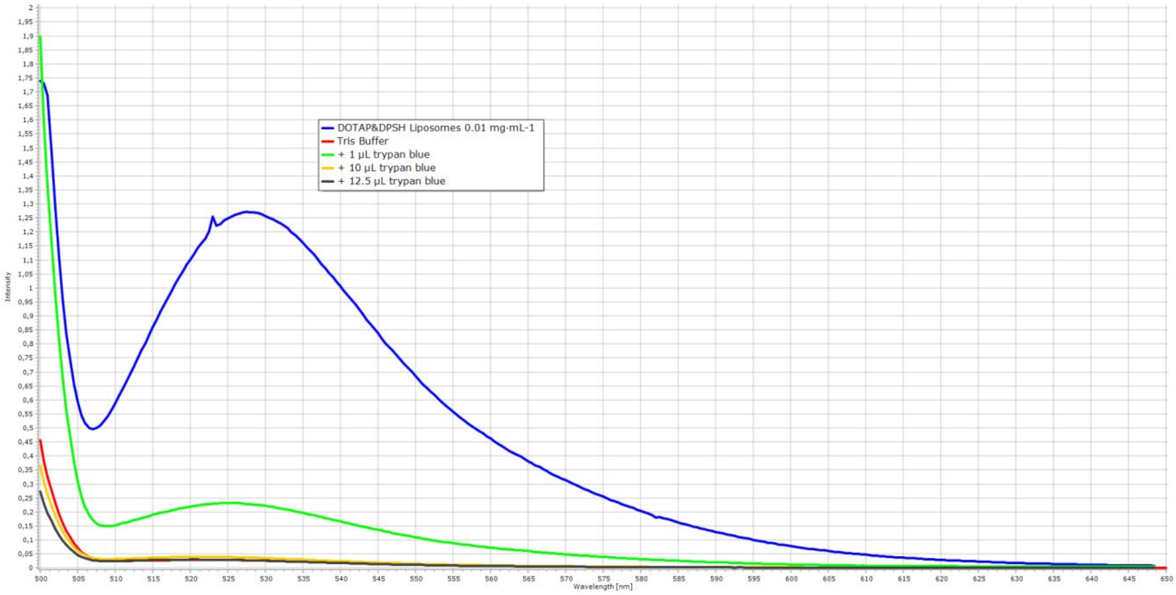

Figure S3. *Decrease in fluorescence intensity of DOTAP&DPSH Liposomes upon gradual addition of 4.0% trypan blue solution. The lipid concentration was selected to correspond to the lipid concentration in the cell culture medium during internalization experiments using flow cytometry.*

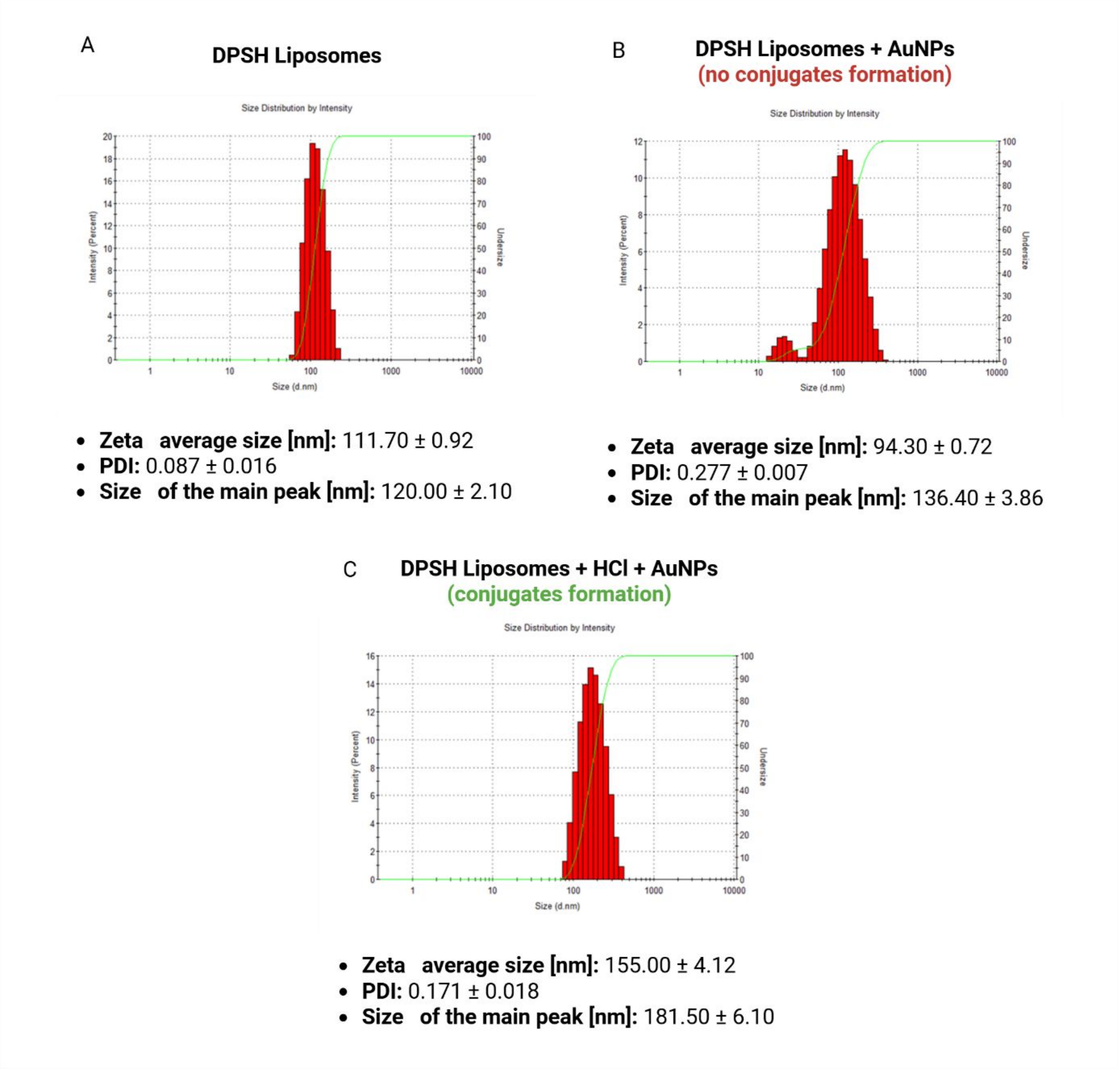

Figure S4. *Comparison of Size Distribution Charts of selected nanosystems obtained by the dynamic light scattering method.* ***(A)*** *Bare DPSH Liposomes.* ***(B)*** *DPSH Liposomes after addition to AuNPs solution, two distinct peaks indicate that two major fractions of nanoobjects are present in the solution and LipoAuNPs conjugates are not properly form.* ***(C)*** *Acidified DPSH Liposomes after addition to AuNPs solution, the shift of peak indicates LipoAuNPs conjugates formation.*

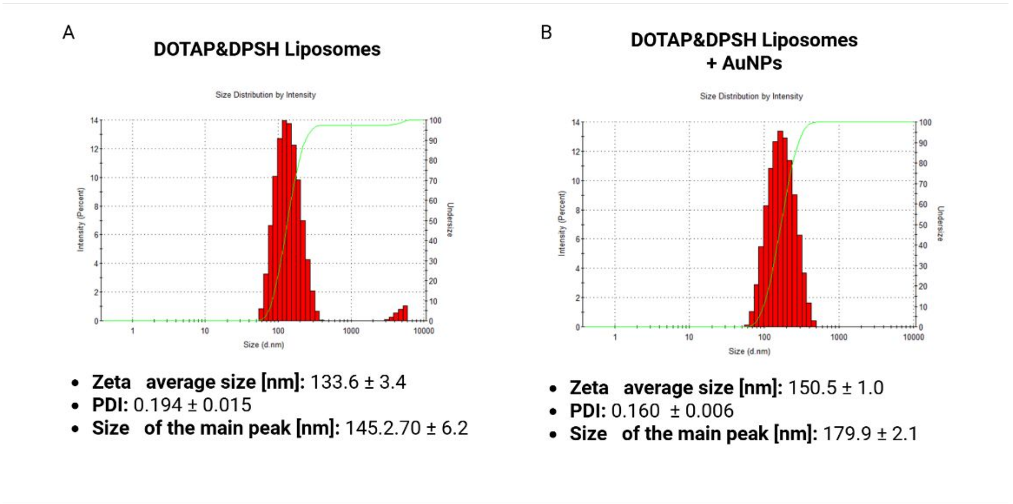

Figure S5. *Comparison of Size Distribution Charts of selected nanosystems obtained by the dynamic light scattering method.* ***(A)*** *Bare DOTAP&DPSH Liposomes.* ***(B)*** *DOTAP&DPSH Liposomes after addition to AuNPs solution, the shift of peak indicates LipoAuNPs conjugates formation.*

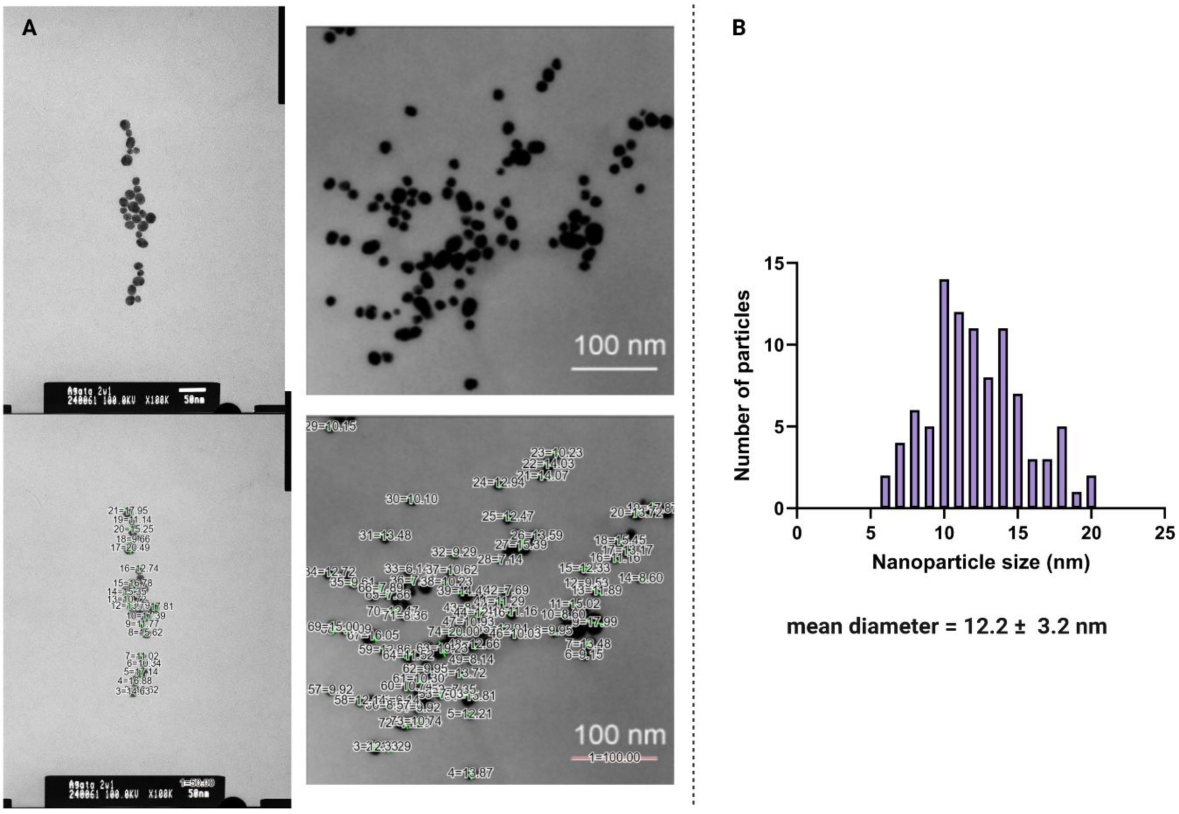

Figure S6. ***(A)*** *Transmission electron microscopy (TEM) micrographs of citrate-AuNPs. Top-left: Original TEM micrographs taken at two different magnifications. Bottom-left: Same images with measured particle diameters overlaid, using the scale bars and the photo measure tool (https://eleif.net/photomeasure). These measurements were used to determine the size distribution of the nanoparticles. The average particle size was calculated based on measurements of 94 individual NPs.* ***(B)*** *Histogram of AuNPs diameters (n = 94), measured from TEM micrographs. Mean = 12.2 nm; SD = 3.2 nm.*

A. Free AuNPs

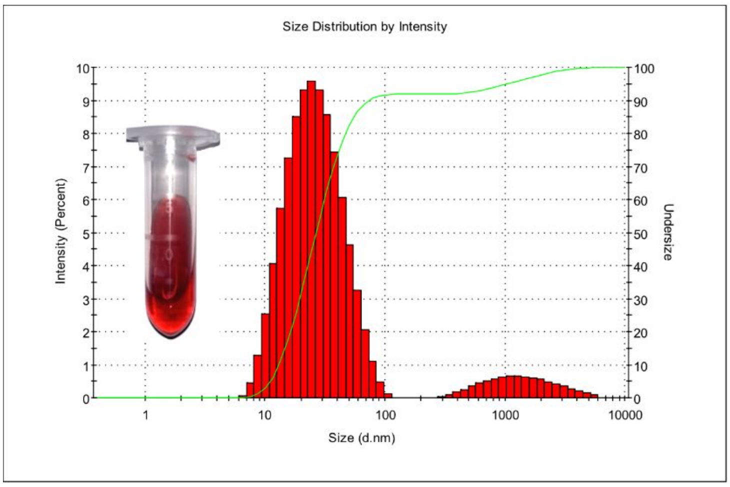

**Zeta average size [nm]**	24,89 ± 0,99
**PDI**	0,343 ± 0,013
**Number mean [nm]**	13,31 ± 0,74
**Size of the main peak [nm]**	28,73 ± 2,11

Acidification with HCl

B. AuNPs after acidifications

(conditions corresponding to those used for AuNPs attachment to DPSH Liposomes)

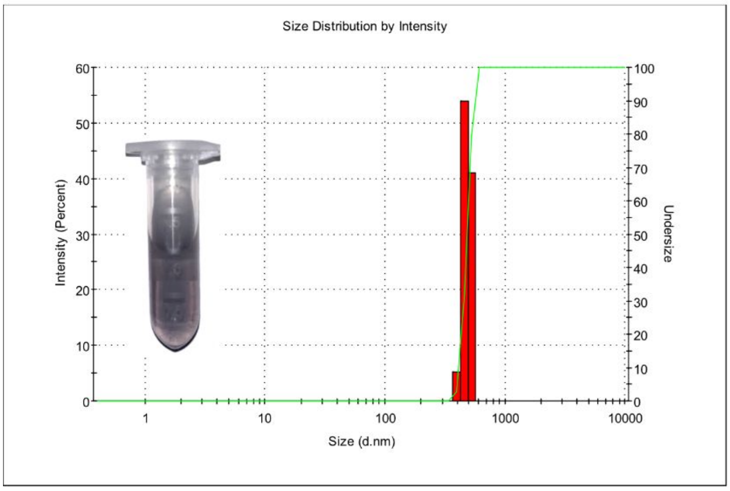

**Zeta average size [nm]**	1682 ± 181
**PDI**	0,820 ± 0,135
**Number mean [nm]**	488,8 ± 60,2
**Size of the main peak [nm]**	493,5 ± 62,6

Figure S7. *Control experiment assessing whether the observed increase in hydrodynamic diameter for DPSH liposomes could be attributed to AuNP aggregation induced by acidic pH. (A) Free citrate-stabilized AuNPs. (B) Citrate-stabilized AuNPs after acidification.*

EDX analysis of a gold-coated surface deposited on a copper substrate after incubation with:

A) DPSH & DOPC Liposomes

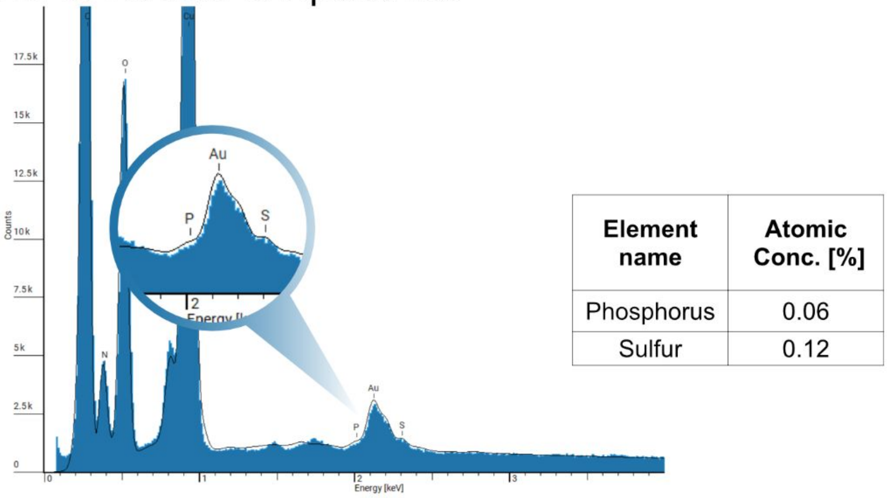

Element name	Atomic Conc. [%]
Phosphorus	0.06
Sulfur	0.12

B) DOPC Liposomes

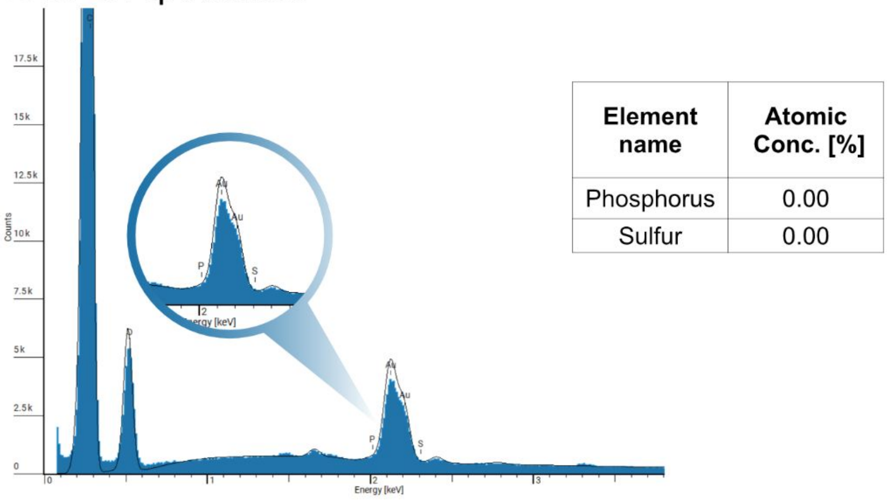

Element name	Atomic Conc. [%]
Phosphorus	0.00
Sulfur	0.00

Figure S8. *Elemental composition determined by EDX analysis of gold-coated copper wires after incubation with liposomes composed of DOPC supplemented with 10 wt% DPSH (A) or pure DOPC (B).*

It should be noted that the liposomes are located exclusively on the surface of the sample, while EDX probes the material to a depth of several micrometers while the thickness of the lipid-based deposit will have up to around 100 nm The use of Cu wires sputtered with Au was not accidental as our aim was to increase the analyzed surface (for planar surfaces the signal will be entirely dominated by the support). Therefore, this analysis should only be considered as qualitative study and one should have in mind that these results are very close to the instrumental limits of detection. For this reason, this method should be regarded as complementary rather than definitive evidence.

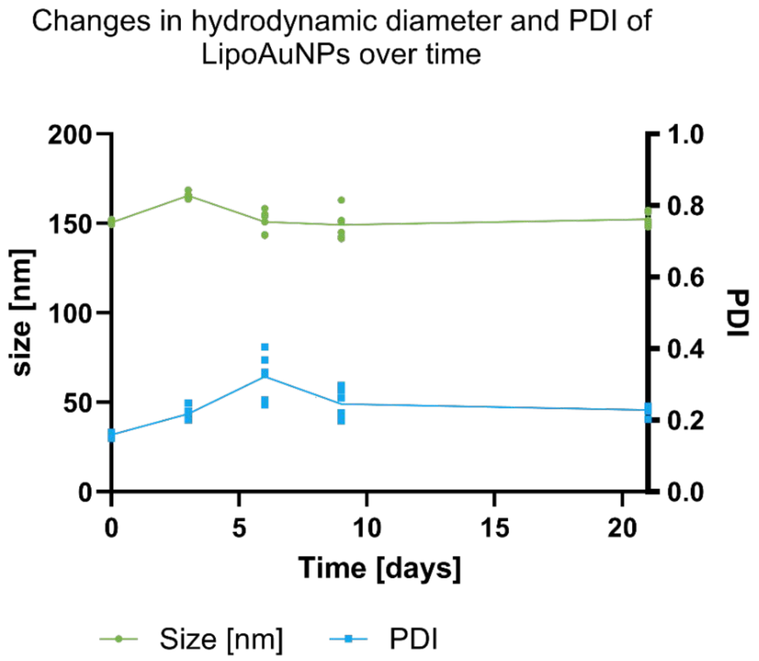

Figure S9. *Changes in hydrodynamic diameter (left y axis) and PDI (right y axis) of LipoAuNPs over time starting from the day of synthesis. LipoAuNPs were diluted 10-fold for measurements. Between measurements, the sample was stored in a measuring cuvette covered with parafilm at a temperature of 4°C. Each measurement was performed in six replicates.*

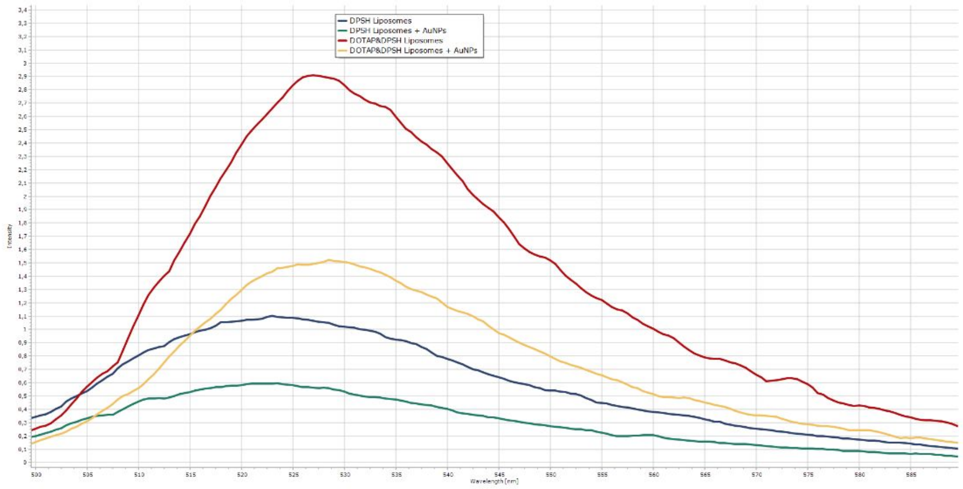

Figure S10. *Fluorescence emission spectra of carboxyfluorescein-labeled liposomes measured at an excitation wavelength of 490 nm. For measurement, each type of liposome was diluted to a lipid concentration of 0.125 mg · ml^{-1} in Tris 10 mM solution. Differences in emission intensity reflect different organization of lipids in the lipid bilayer and partial fluorescence quenching caused by the presence of gold nanoparticles. Additionally, the difference between bare DPSH Liposomes and DOTAP&DPSH Liposomes is the result of their difference in surface charge. Since, DPSH Liposomes are negatively charged they more strongly attract H^+ ions which leads to carboxyfluorescein protonation and eventually to decrease in fluorescence level.*

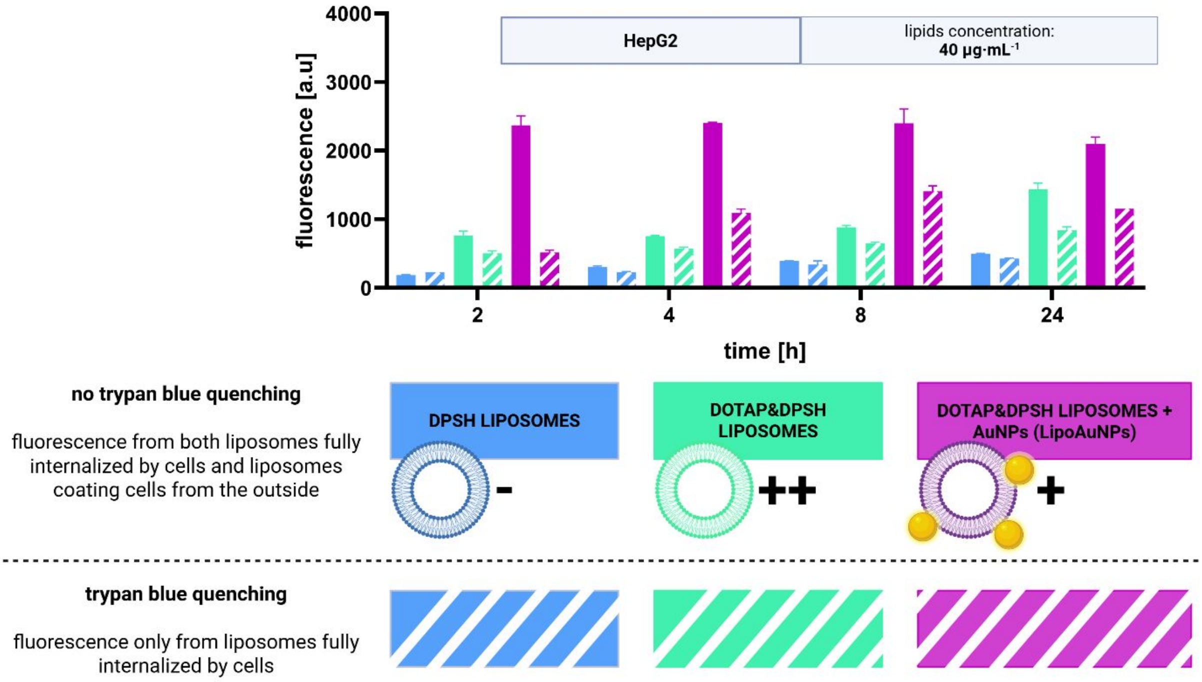

Figure S11. *Comparison of cell fluorescence with and without trypan blue external fluorescence quenching for cellular uptake measurements by flow cytometry for HepG2 cells. Cells were incubated for 2, 4, 8 and 24 h with DPSH liposomes, DOTAP&DPSH liposomes and DOTAP&DPSH liposomes with AuNPs. All results are presented as the average of two independent measurements ± standard deviation. Each measurement is an average of 10000 cells. Results are presented as mean fluorescence for lipids concentration of 40 µg·mL*$^{-1}$.

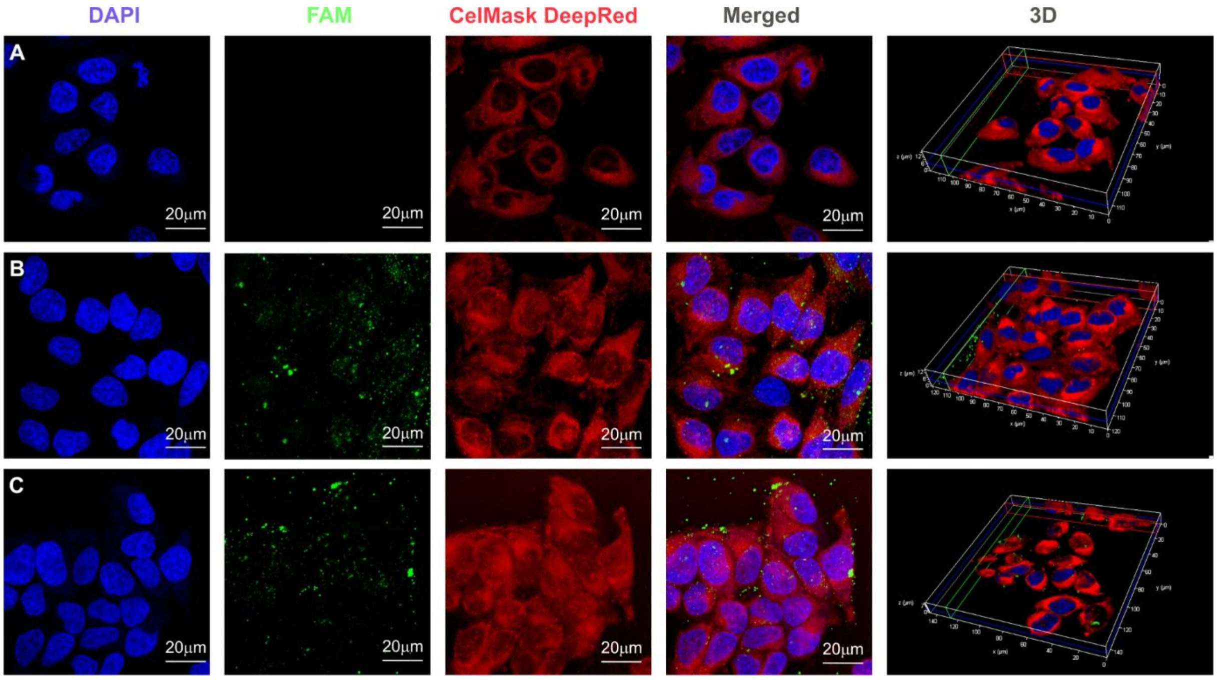

Figure S12. *Confocal images of untreated HeLa cells (**A**) and HeLa treated with DOTAP&DPSH liposomes (**B**) and DOTAP&DPSH liposomes with gold nanoparticles (**C**). Cells were incubated with liposomes 40 µg·mL^{-1} for 4 hours.*

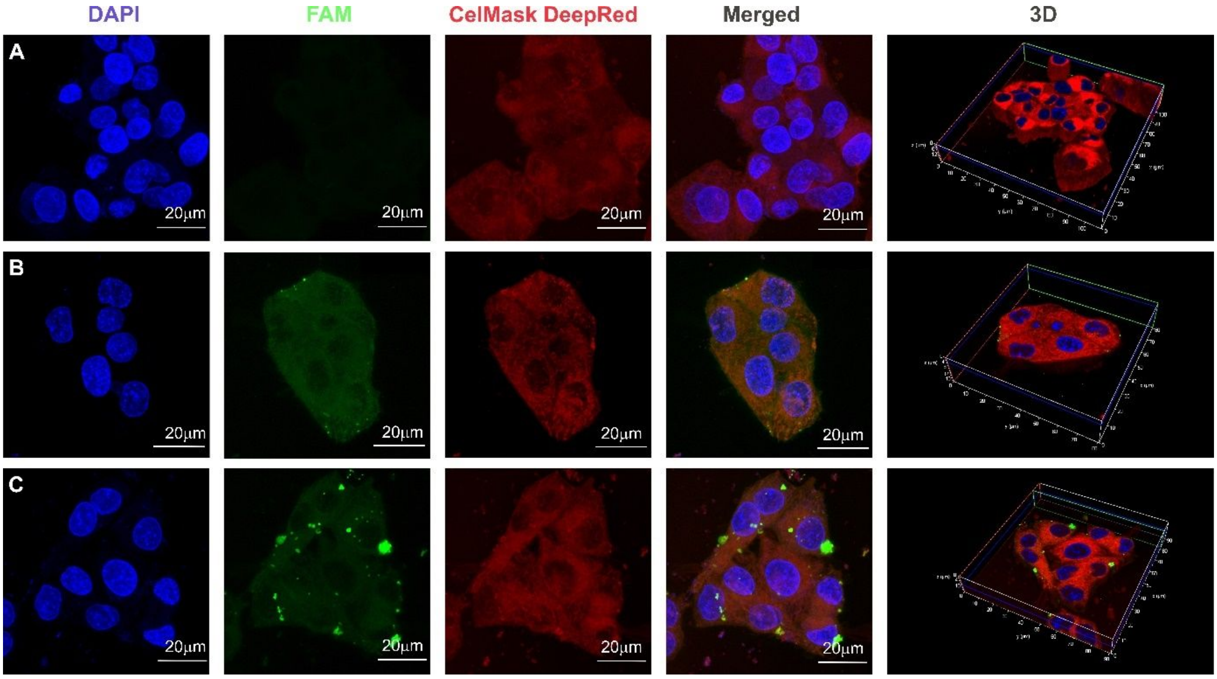

Figure S13. *Confocal images of untreated HepG2 cells (**A**) and HepG2 treated with DOTAP&DPSH liposomes (**B**) and DOTAP&DPSH liposomes with gold nanoparticles (**C**). Cells were incubated with liposomes 40 µg·mL^{-1} for 4 hours.*

Table S1. *Statistical analysis results. Two-way ANOVA analysis was performed to compare the effect of lipid concentration and liposome type on cytotoxicity against HeLa and HepG2 cell lines. To assess the effect of charge on cytotoxicity and the effect of AuNPs attachment, DPSH Liposomes and DOTAP& DPSH Liposomes, as well as DOTAP& DPSH Liposomes and DOTAP& DPSH Liposomes with AuNPs, were compared. For this purpose, multiple comparisons were performed within the two-way ANOVA framework with Šídák's multiple comparisons test for both cell lines.*

Analyzed parameter	*HeLa*	*HepG2*
Two-way Anova		
Lipids concentration	**** ($p < 0.0001$)	*No statistically significant difference*
Liposome type	* ($p = 0{,}0141$)	*No statistically significant difference*
Multiple comparisons within the two-way ANOVA framework Šídák's multiple comparisons test		
DPSH Liposomes vs DOTAP& DPSH Liposomes (influence of charge)	*No statistically significant difference*	*No statistically significant difference*
DOTAP& DPSH Liposomes vs DOTAP& DPSH Liposomes with AuNPs (influence of modification with gold nanoparticles)	*No statistically significant difference*	*No statistically significant difference*

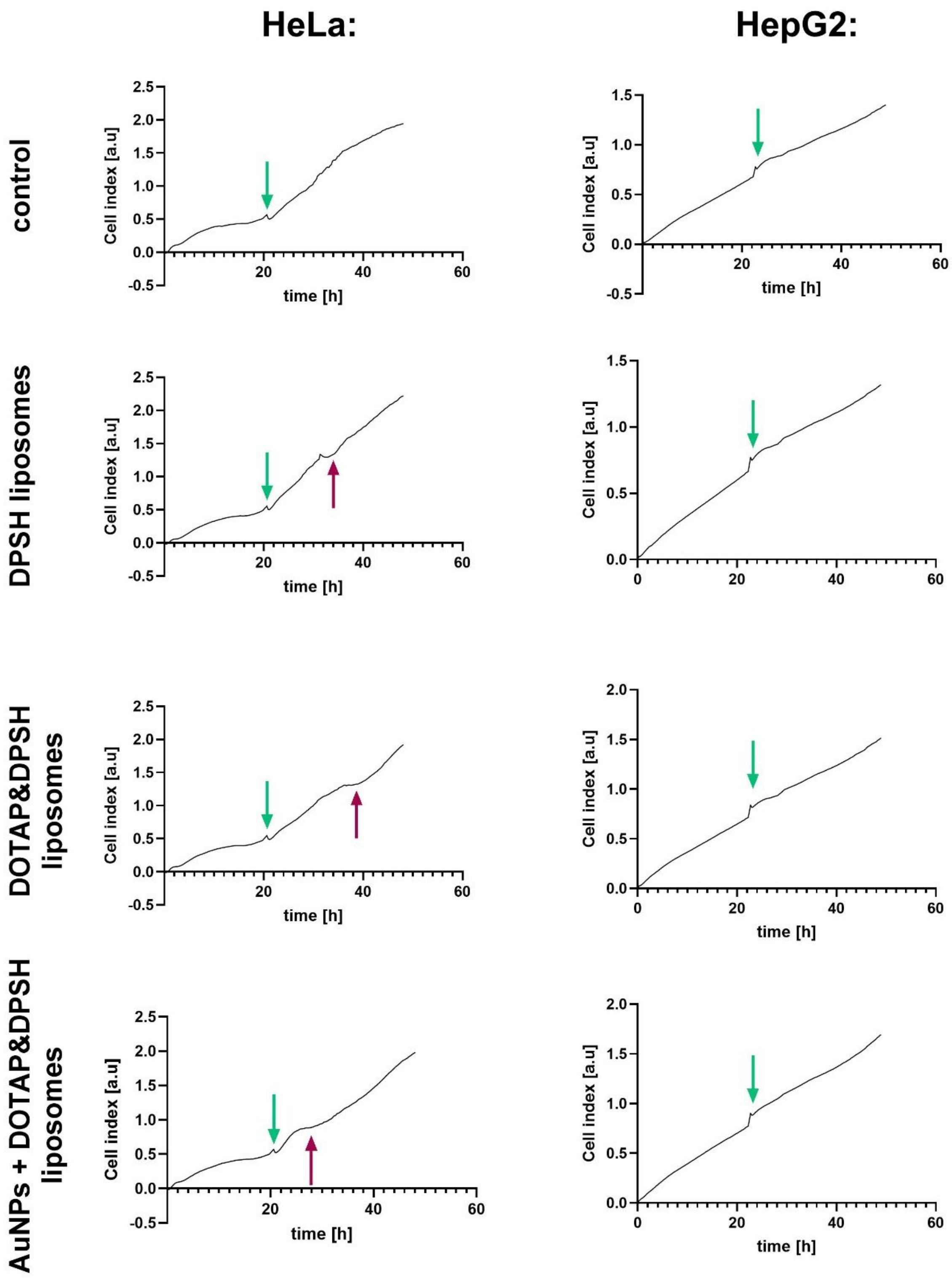

Figure S14. *Real-time monitoring of cell viability of the HeLa and HepG2 cell line after incubation with LipoAu. Cells with liposomes at 200 µg·mL*$^{-1}$ *were monitored in four wells. After the experiment, cell index values were averaged.* ***Green arrow indicates the moment of liposome addition. Red arrow indicates the moment of proliferation inhibition.***
